# Sonodynamic therapy augmented by glycolysis inhibition: a novel metabolic reprogramming strategy for enhanced osteosarcoma treatment

**DOI:** 10.1093/nsr/nwaf365

**Published:** 2025-08-30

**Authors:** Zhuorun Song, Shunyi Lu, Yuqi Yang, Zijun Chen, Youdong Chen, Jie Cao, Zimin Zhang, Jun Ge, Huilin Yang, Liang Cheng

**Affiliations:** Department of Orthopedic Surgery, The First Affiliated Hospital of Soochow University, Suzhou 215006, China; Institute of Functional Nano & Soft Materials (FUNSOM), Jiangsu Key Laboratory for Carbon-Based Functional Materials & Devices, Soochow University, Suzhou 215123, China; Department of Orthopedic Surgery, The First Affiliated Hospital of Soochow University, Suzhou 215006, China; Institute of Functional Nano & Soft Materials (FUNSOM), Jiangsu Key Laboratory for Carbon-Based Functional Materials & Devices, Soochow University, Suzhou 215123, China; Institute of Functional Nano & Soft Materials (FUNSOM), Jiangsu Key Laboratory for Carbon-Based Functional Materials & Devices, Soochow University, Suzhou 215123, China; Department of Breast Surgery, Obstetrics & Gynecology Hospital of Fudan University, Yangtze River Delta Integration Demonstration Zone (Qingpu), Shanghai 201799, China; Institute of Functional Nano & Soft Materials (FUNSOM), Jiangsu Key Laboratory for Carbon-Based Functional Materials & Devices, Soochow University, Suzhou 215123, China; Institute of Functional Nano & Soft Materials (FUNSOM), Jiangsu Key Laboratory for Carbon-Based Functional Materials & Devices, Soochow University, Suzhou 215123, China; Department of Orthopedic Surgery, The First Affiliated Hospital of Soochow University, Suzhou 215006, China; Department of Orthopedic Surgery, The First Affiliated Hospital of Soochow University, Suzhou 215006, China; Institute of Functional Nano & Soft Materials (FUNSOM), Jiangsu Key Laboratory for Carbon-Based Functional Materials & Devices, Soochow University, Suzhou 215123, China; Department of Orthopedic Surgery, The First Affiliated Hospital of Soochow University, Suzhou 215006, China; Institute of Functional Nano & Soft Materials (FUNSOM), Jiangsu Key Laboratory for Carbon-Based Functional Materials & Devices, Soochow University, Suzhou 215123, China

**Keywords:** osteosarcoma, glycolysis inhibition, sono-immune strategy, immune activation, immunosuppression reversion

## Abstract

A metabolic reprogramming strategy, considered an efficient way to enhance current therapies, has provided renewed hope for treating osteosarcoma (OS), which has reached a bottleneck in clinical practice. In this study, SHK@Mn-TiO_2_ were developed as novel sonodynamic therapy (SDT) agents with glycolysis-inhibiting properties. By reducing the expression of pyruvate kinase isozyme M2 (PKM2) and hexokinase-2 (HK-2), SHK@Mn-TiO_2_ effectively inhibited glycolysis, thereby reversing the hypoxic tumor microenvironment (TME), as evidenced by a more than ∼50% decrease in hypoxia-inducible factor-1α (HIF-1α) and lactate (LA) levels compared with those of Mn-TiO_2_. Under this O_2_-enriched TME, SHK@Mn-TiO_2_ enhanced intracellular reactive oxygen species (ROS) levels by ∼53% and increased K7M2 tumor inhibition under ultrasound (US). Furthermore, the combination of glycolysis inhibition and SDT initiated a cascade of immune responses, promoting an ∼98% increase in the maturation of dendritic cells and ∼280% increase in the infiltration of IFN-γ^+^ CD8^+^ T cells compared with those in the control. The typically immunosuppressive TME induced by conventional SDT was significantly reversed, as indicated by the reduction in the proportions of regulatory T cells to ∼18% and myeloid-derived suppressor cells (MDSCs) to ∼49% in the Mn-TiO_2_ groups. Moreover, a long-term immune memory effect was observed in the murine osteosarcoma cell line (K7M2) tumor rechallenge model as a result of strong immune activation. Overall, this study highlights a sono-immune strategy for OS treatment based on the synergistic effects of glycolysis inhibition combined with SDT, offering a promising solution to the current therapeutic challenges in clinical OS management.

## INTRODUCTION

Osteosarcoma (OS) is considered the most prevalent primary malignant bone tumor, predominantly affects children and adolescents, and has emerged as a significant global public health concern [1]. Despite comprising only 15% of all extracranial solid tumors in this age group, its aggressive characteristics contribute to poor long-term survival rates and negatively impact patient outcomes and quality of life [2]. The introduction of neoadjuvant chemotherapy combined with surgery since the 1970s has notably improved the 5-year event-free survival rates for OS patients [3,4]. Unfortunately, survival rates for recurrent OS have improved a little over the past 30 years [5]. Recent studies highlight the exceptionally unstable molecular landscape of OS, which is characterized by profound intra- and inter-tumor heterogeneity, few recurrent targetable mutations, and extensive tumor variability. These factors have severely hindered the development of targeted therapies and are key contributors to the poor clinical outcomes of OS patients [6,7].

Recently, the shift toward aerobic glycolysis has been recognized as a common trait of tumor cells [8–11]. Accumulating evidence indicates that glycolysis can facilitate tumor cell immune evasion through multiple mechanisms [12]. In brief, the competition for energy resources directly impacts cellular functions. First, T cells rely on glycolysis for antitumor activity and are significantly suppressed due to the avid glucose uptake of tumor cells [13]. Furthermore, aerobic glycolysis byproducts, such as lactate (LA), inhibit T-cell proliferation and function by suppressing their glycolytic capabilities [14,15]. In addition, the hypoxic tumor microenvironment (TME) generated by aerobic glycolysis further impairs T cells [16–18]. Finally, the aerobic glycolysis pathway in tumors can directly regulate the expression of various immunomodulatory factors, circumventing innate immune suppression against tumors [12,19–21]. Therefore, inhibiting aerobic glycolysis to block tumor energy and reverse the immunosuppressive TME has emerged as a critical approach to enhancing T-cell function and has demonstrated significant efficacy in preventing tumor growth.

Medical ultrasound plays a pivotal role in modern cancer diagnosis and therapy because of its excellent biological effects [22–26]. Sonodynamic therapy (SDT), a novel cancer treatment strategy that combines sonosensitizers with ultrasound (US), has tremendous potential for treating deep-seated tumors owing to its unparalleled tissue penetration capabilities [23,27–33]. Specifically, US frequencies between 20 kHz and 3 MHz are employed in current SDT applications. Operating at low intensity, US achieved significant tissue penetration depths of ∼10 cm, enabling it to reach deeper target sites [22]. A range of high-performance inorganic multifunctional SDT nanoplatforms have been developed, demonstrating outstanding capabilities for reactive oxygen species (ROS) generation, drug delivery, and the induction of immunogenic cell death (ICD) [30,34–40]. However, the therapeutic efficacy of SDT can be diminished by tumor-mediated immune resistance triggered as a self-protective mechanism [41]. Tunable inorganic nanoplatforms have outstanding ability to carry and release various inorganic ions after material engineering, offering a promising avenue for modulating TME immunity to enhance therapeutic outcomes [42–45]. Thus, strategically enhancing SDT efficacy represents a pivotal yet pressing scientific challenge in the realm of cancer therapy.

**Scheme 1.**
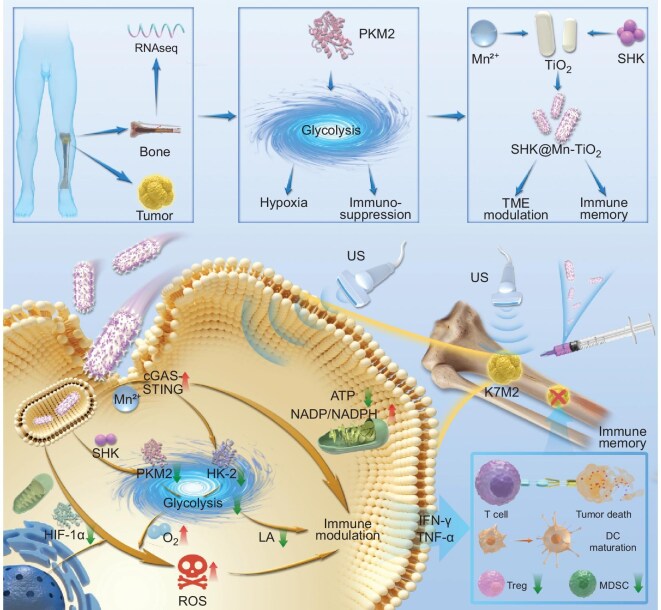
SHK@Mn-TiO_2_ was designed and synthesized on the basis of its high level of OS glycolysis according to bioinformatics analysis. Upon US irradiation, SHK@Mn-TiO_2_ generated ROS and released debris to stimulate DC maturation to further activate anticancer immunity, thereby inhibiting outstanding K7M2 proliferation and promoting immune cell recruitment.

Herein, we developed a simple but efficient sonodynamic-immunotherapy nanoplatform, SHK@Mn-TiO_2,_ by combining the high-performance sonosensitizer Mn-TiO_2_ with shikonin (SHK), an inhibitor of the key glycolytic enzyme pyruvate kinase isozyme M2 (PKM2), which is specifically tailored to address the unique biological features of bone for OS treatment (Scheme 1). Upon SHK@Mn-TiO_2_ delivery into OS cells (K7M2), SHK significantly inhibited glycolysis by suppressing PKM2 expression. Additionally, the rapid release of Mn^2+^ activated the cyclic GMP-AMP synthase (cGAS)–stimulator of interferon genes (STING) pathway, leading to the reduction of another key glycolytic enzyme, hexokinase-2 (HK-2), thereby further inhibiting glycolysis. Dual glycolysis inhibition increases the level of intracellular O_2_, reduces the expression of hypoxia-inducible factor-1α (HIF-1α), reverses the hypoxic TME, and decreases lactate secretion. Moreover, mitochondrial dysfunction further suppresses ATP production. Enhanced intracellular O_2_ amplifies the sonodynamic effect of Mn-TiO_2_, enabling the generation of ROS under US irradiation, inducing ICD and further disrupting ATP production. Owing to the combined effects of cGAS-STING pathway activation and glycolysis inhibition, the immunosuppressive TME induced by conventional SDT was significantly reversed. Importantly, tumor cells exhibited increased secretion of interferon-γ (IFN-γ) and tumor necrosis factor-α (TNF-α), leading to cytotoxic T lymphocytes (CTLs) activation and dendritic cells (DCs) maturation, accompanied by a reduction in the number of immunosuppressive regulatory T cells (Tregs) and myeloid-derived suppressor cells (MDSCs). Both *in vitro* and *in vivo* studies demonstrated that this nanoplatform potently inhibited tumor growth and significantly extended the survival of OS-bearing mice without evident systemic toxicity. A long-term immune memory effect was also observed in the K7M2 tumor rechallenge model as a result of strong immune activation.

## RESULTS AND DISCUSSION

### Aerobic glycolysis and tumor immune evasion in OS

**Figure 1.**
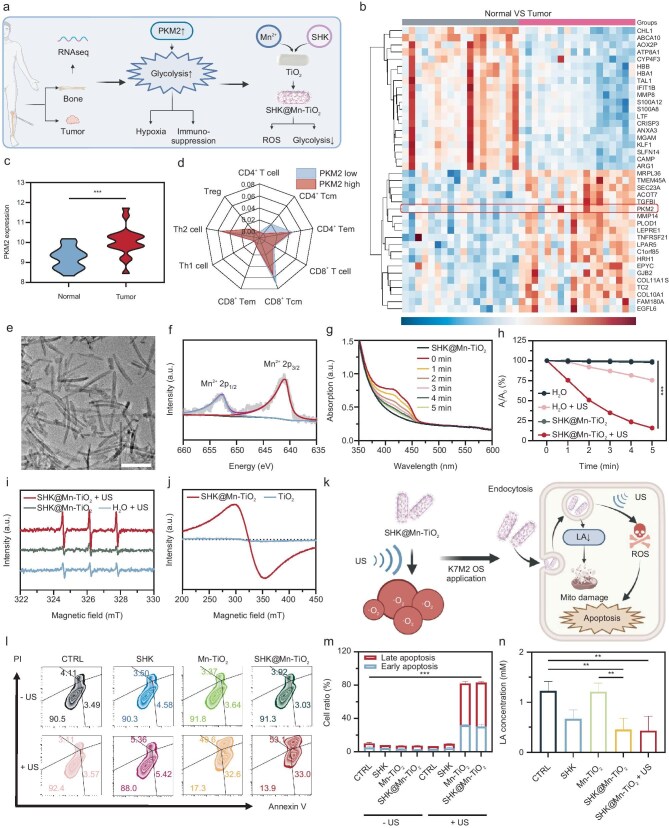
Glycolysis in OS, characterization of SHK@Mn-TiO_2_ and evaluation of its therapeutic effect on K7M2 cells. (a) Scheme of the bioinformatics analysis of glycolysis in OS patients. (b) Heat map of DEGs between normal bones and OS tissues. (c) Box plot of PKM2 expression in normal bone and OS tissues. (d) Estimated proportions of T cells in different OS tissues divided by PKM2 expression. (e) TEM images of SHK@Mn-TiO_2_. Scale bar  =  50 nm. (f) XPS spectra of Mn 2p. (g) Time-dependent changes in the DPBF absorption of SHK@Mn-TiO_2_ under US irradiation. (h) Comparison of the sonodynamic performance of H_2_O, H_2_O+US, SHK@Mn-TiO_2_, and SHK@Mn-TiO_2_+US. (i) ESR spectra of H_2_O+US, SHK@Mn-TiO_2_, and SHK@Mn-TiO_2_+US obtained by using TEMP as the probe. (j) Comparison of the cavitation effects of TiO_2_ and SHK@Mn-TiO_2_. (k) Scheme of the sonodynamic effect induced by SHK@Mn-TiO_2_ and its application in K7M2 cells. Representative flow dot plots (l) and quantitative analysis (m) of cell apoptosis after different treatments. (n) LA concentration caused by glycolysis inhibition after different treatments. All values are shown as means  ±  SD (*n*  =  3) *P < 0.05, **P < 0.01, ***P < 0.001.

Aerobic glycolysis serves as a critical energy source for tumor cells, fostering their proliferation and immune evasion [46]. Analysis of genetic data from the Gene Expression Omnibus (GEO) database revealed numerous differentially expressed genes (DEGs) related to OS (Fig. 1a), with significant upregulation of key glycolytic enzyme PKM2-related genes (Figs 1b, c, and S1). Immunofluorescence confirmed higher PKM2 expression in OS cells than in mouse macrophages (RAW 264.7) (Fig. S2a). By shifting our focus to the metabolic milieu, we quantified LA, a key byproduct of tumor glycolysis, with an LA quantitative assay. LA levels were prominently elevated in OS, both in murine- and human-derived cell lines (K7M2 cells and MG-63 human osteosarcoma cells), compared with normal cells (RAW 264.7 (murine macrophage-like cell line) and HUVECs (human umbilical vein endothelial cells)) (Fig. S2b). To investigate the impact of glycolysis levels on immune cells in the OS TME, OS samples were divided into low and high groups according to PKM2 expression, and then immune infiltration analysis was conducted via xCell webtool. Higher PKM2 expression is associated with reduced CD4^+^ and CD8^+^ T cells and increased Tregs, highlighting the role of glycolysis in immune suppression (Figs 1d and S3). Moreover, the functional changes in immune cells induced by glycolysis might be interrelated, potentially playing a critical role in exacerbating tumor immune suppression (Fig. S4). These findings underscored the strong correlation between increased glycolysis, which was characterized by PKM2 overexpression, and the suppression of antitumor immunity in OS. Furthermore, these findings suggested a complex interplay wherein glycolysis-induced functional alterations in immune cells may reciprocally reinforce, potentially exacerbating the immunosuppressive TME. Collectively, our findings underscored the strategic importance of targeting aerobic glycolysis as a means to disrupt this vicious cycle and revitalize antitumor immune responses in OS.

### SHK@Mn-TiO_2_ nanoplatform: synthesis, characterization, and sonodynamic effects

Small molecule glycolysis inhibitors are rapidly metabolized and eliminated from tumor cells because of their inherently short half-life after their cellular uptake [36]. On the other hand, inorganic nanomaterials exhibit remarkable advantages in safeguarding drugs from degradation, prolonging their intracellular retention, and simultaneously offering ion-mediated biological modulation and therapeutic capabilities [47]. Considering the unique characteristic of bone tissue as the primary hard component, we selected Mn^2+^-doped TiO_2_, which was renowned for its excellent SDT performance, as the delivery vehicle for the PKM2 inhibitor SHK. The synthesis of Mn-TiO_2_ was achieved via the classical high-temperature thermal decomposition method, which employed MnCl_2_ and TiCl_4_ as precursors. Subsequently, SHK with ∼2% Mn^2+^ content was incorporated through 1,2-distearoyl-sn-glycero-3-phosphoethanolamine-modified polyethylene glycol (MW  =  2000, DSPE-PEG2000) surface modification by the hydrophobic effect, resulting in the final water-soluble SHK@Mn-TiO_2_ nanoplatform, which was tailored for biological applications (Fig. S5). Transmission electron microscopy (TEM) imaging revealed the rod-like morphology of SHK@Mn-TiO_2_ with a length of ∼100 nm (Fig. 1e). Elemental mapping and energy dispersive spectroscopy (EDS) confirmed the presence of Mn and Ti atoms within these nanorods (Fig. S6a, b). X-ray powder diffraction (XRD) confirmed a crystalline structure corresponding to TiO_2_, with no significant alterations observed upon Mn^2+^ doping (Fig. S7). Furthermore, X-ray photoelectron spectroscopy (XPS) analysis refined the valence states of Mn and Ti (Figs 1f and S8a, b). In the Mn 2p spectrum, the binding energies of Mn (II) 2p_3/2_ and Mn (II) 2p_1/2_ were identified at 640.98 eV and 652.78 eV, respectively, while the binding energies of Ti (IV) 2p_3/2_ and Ti (IV) 2p_1/2_ were observed at 458.48 eV and 464.18 eV in the Ti 2p spectrum, respectively. Dynamic light scattering (DLS) analysis revealed a relatively uniform hydrodynamic diameter of ∼30 nm for SHK@Mn-TiO_2_ dispersed in water (Fig. S9), further underscoring its suitability for biological applications. Stability assessments under water and simulated physiological conditions over 7 days demonstrated remarkable stability (Fig. S10), indicating the robustness of the constructed nanoplatform.

TiO_2_ nanoparticles have been widely used as an excellent sono-sensitizer for SDT [48]. First, the US used in the subsequent experiments was determined at 3 W/cm^2^ for 3 min, based on the mechanical damage effect of US on K7M2 cells (Fig. S11). To investigate the sonodynamic effects, a ROS-specific fluorescent probe, 1,3-diphenylisobenzofuran (DPBF), was utilized to monitor ROS generation by SHK@Mn-TiO_2_ under US irradiation. As anticipated, SHK@Mn-TiO_2_ exhibited time-dependent ROS generation under US irradiation (Fig. 1g, h). Electron spin resonance (ESR) measurements were further conducted to investigate the generation of singlet oxygen (^1^O_2_). The ESR spectrum exhibited a characteristic peak at a ratio of 1:1:1 when 2,2,6,6-tetramethyl-4-piperidinone (TEMP) was used as a standard detection probe, indicating the ^1^O_2_ generation of SHK@Mn-TiO_2_ under US irradiation (Fig. 1i). Moreover, cavitation effects contributed to an enhanced reduction in DPBF absorption, further corroborating the sonodynamic efficacy (Fig. 1j). Above all, SHK@Mn-TiO_2_ exhibited outstanding sonodynamic efficacy and further supported applications at the cellular level.

The release of inorganic ions plays a critical role in inorganic nanoplatforms. The release kinetics of Mn^2+^ were quantified via inductively coupled plasma-optical emission spectrometry (ICP-OES). Notably, >60% of the Mn^2+^ among SHK@Mn-TiO_2_ was rapidly released within 1 h, indicating efficient ion release for biological modulation (Fig. S12). To assess the Fenton-like activity of Mn^2+^, tetramethylbenzidine (TMB), a hydroxyl radical (·OH)-specific probe, was utilized [28]. The presence of characteristic peaks following incubation with SHK@Mn-TiO_2_, which intensified with increasing H_2_O_2_ concentrations, confirmed the generation of ·OH from Mn^2+^-mediated Fenton-like reactions (Fig. S13). Above all, the SHK@Mn-TiO_2_ nanoplatform had remarkable potential for tumor therapeutic applications by generating substantial amounts of ^1^O_2_ under short-term US exposure and swiftly releasing Mn^2+^ to facilitate Fenton-like reactions, thereby increasing the generation of ·OH. This multifunctional nanoplatform held promise for advancing the frontier of SDT for the treatment of OS.

### Antitumor efficacy of the SHK@Mn-TiO_2_ nanoplatform for OS

Supported by the outstanding ROS generation capacity of SHK@Mn-TiO_2_, we evaluated its *in vitro* antitumor efficacy (Fig. 1k). First, Cy5.5-labeled SHK@Mn-TiO_2_ was prepared and incubated with K7M2 cells. Confocal laser scanning microscopy (CLSM) images confirmed the gradual internalization of SHK@Mn-TiO_2_ by K7M2 cells over 12 h, revealing its biological effects (Fig. S14). To determine their biocompatibility, RAW 264.7 cells were exposed to various concentrations of SHK@Mn-TiO_2_ for 24 h, and methyl thiazolyl tetrazolium (MTT) assays confirmed the excellent safety profile of SHK@Mn-TiO_2_ (Fig. S15).

Encouraged by the above findings, K7M2 cells were subjected to various treatments, revealing that the synergistic combination of SHK@Mn-TiO_2_ and US (SHK@Mn-TiO_2_+US) exhibited a significantly potent cytotoxic effect on K7M2 cells (Fig. S16). Unfortunately, even the lowest intensity of ultrasound allowed by the instrument could still cause mechanical damage to K7M2 cells. This situation might be improved with the advancement of ultrasonic equipment in the future. The distinctive visualization of live and dead cells through co-localization of calcein acetoxymethyl (Calcein AM) and propidium iodide (PI) highlighted a profound reduction in viable cells and a surge in cell death following SHK@Mn-TiO_2_+US treatment (Fig. S17). To assess the degree of apoptosis, a standardized Annexin V/PI assay was conducted, and the results were measured via fluorescence-activated cell sorting (FACS), which revealed a dramatic increase in both early and late apoptosis rates in K7M2 cells upon SHK@Mn-TiO_2_+US treatment (Fig. 1l, m). Moreover, we further observed changes in the expression levels of the apoptosis-related proteins Bcl-2 and Bax, indicating that SHK@Mn-TiO_2_ in conjunction with US effectively induced apoptosis in K7M2 cells (Fig. S18).

Additionally, excessive ROS accumulation triggered mitochondrial damage, as evidenced by alterations in the mitochondrial membrane potential (MMP), which was analyzed via the 5,5,6,6-tetrachloro-1,1,3,3-tetraethylbenzimidazolylcarbocyanine iodide (JC-1) assay [49]. FACS results revealed the conversion of red fluorescent J-aggregates, representing a normal MMP, into green monomers, revealing that MMP disruption is mediated by ROS generation and ultimately leads to cell death (Fig. S19a, b). NADPH in mitochondria typically acts as a reducing agent in biosynthesis, transferring H^+^ to NAD^+^ via enzymatic action, thereby influencing the oxidative phosphorylation process [50]. Notably, the decreased NADP^+^/NADPH ratio in the SHK@Mn-TiO_2_+US group attests to NADPH accumulation resulting from impaired hydrogen transfer, implying compromised oxidative phosphorylation (OXPHOS) and irreversible mitochondrial dysfunction (Fig. S20). Moreover, owing to the excellent glycolysis inhibitory effect of SHK, we observed an ∼63% decrease in LA levels after SHK@Mn-TiO_2_ treatment compared with those of the Mn-TiO_2_ group, emphasizing the successful suppression of intracellular glycolysis (Fig. 1n). In contrast, Mn-TiO_2_ alone caused a slight decrease in LA with no significant statistical difference, whereas US treatment alone had no direct effect on LA secretion. Overall, SHK@Mn-TiO_2_ energized by US irradiation effectively induced cytotoxicity in K7M2 OS cells, suppressed mitochondrial function, and reduced LA secretion, highlighting its promise as a multifaceted antitumor therapeutic approach.

### Enhanced sonodynamic therapy and glycolysis inhibition effects of the SHK@Mn-TiO_2_ nanoplatform for OS

**Figure 2.**
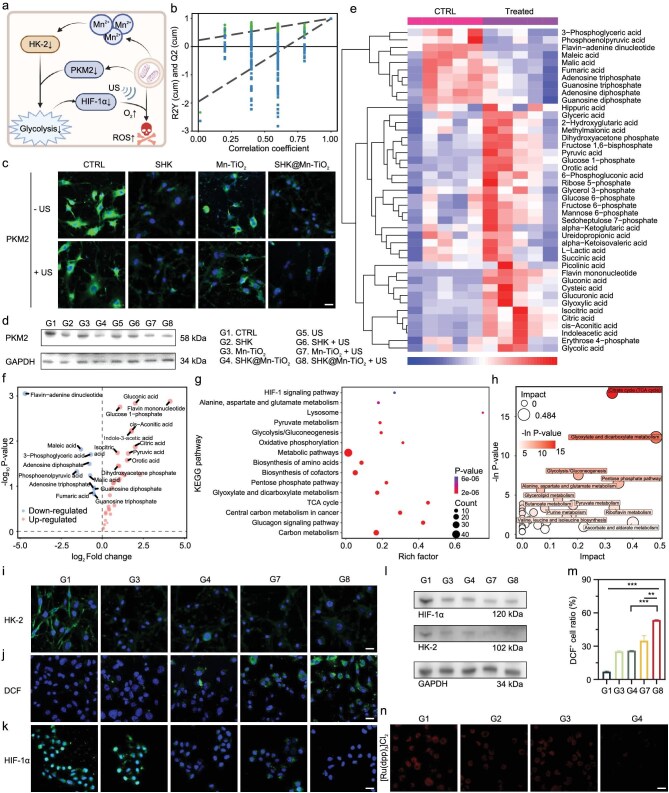
Glycolysis inhibition and the potential mechanisms combined with enhanced SDT. (a) Scheme of glycolysis inhibition combined with SDT caused by SHK@Mn-TiO_2_+US. (b) Permutation plot test of the orthogonal partial least squares discriminant analysis (OPLS-DA) model for the control group (phosphate buffered saline, PBS) vs the treatment group (SHK@Mn-TiO_2_+US). (c) CLSM images of PKM2-specific immunofluorescence after different treatments. Scale bar  =  20 μm. (d) Western blot analysis of PKM2. Groups 1–8 represent CTRL, SHK, Mn-TiO_2_, SHK@Mn-TiO_2_, US, SHK+US, Mn-TiO_2_+US, and SHK@Mn-TiO_2_+US, respectively. Heatmap of hierarchical clustering analysis (e), volcano plot (f), KEGG enrichment bubble (g), and pathway analysis (h) for the control group (PBS) vs treatment group (SHK@Mn-TiO_2_+US). CLSM images of HK-2- (i), DCF- (j), and HIF-1α-specific (k) immunofluorescence after different treatments. Scale bar  =  20 μm. (l) WB analysis of HK-2 and HIF-1α expression after different treatments. SHK@Mn-TiO_2_, (m) Quantitative analysis of the DCF^+^ cell ratio by FACS after different treatments. (n) CLSM images of K7M2 cells stained with the [Ru(dpp)_3_]Cl_2_ probe after different treatments. Scale bar  =  20 μm. All values are shown as the means  ±  SD (*n*  =  3) *P < 0.05, **P < 0.01, ***P < 0.001.

We further explored the potential synergistic mechanisms that underpin the antitumor efficacy of combining glycolysis inhibition with SDT in the treatment of OS. As depicted in Fig. 2a, we revealed the intricate interplay between these therapeutic strategies. First, to investigate the impact of SHK@Mn-TiO_2_ on cell metabolism, we performed comprehensive central carbon metabolism sequencing (Figs 2b and S21). The results revealed the reliable quantification of various metabolites post-treatment. Heatmaps and volcano plots revealed a significant reduction in the level of glycolytic intermediates after SHK@Mn-TiO_2_+US treatment, including 3-phosphoglyceric acid, phosphoenolpyruvic acid, and ATP (Figs 2e, f, and S22). Conversely, there was an increase in metabolites associated with the tricarboxylic acid (TCA) cycle and the pentose phosphate pathway (PPP), suggesting a metabolic shift from highly efficient glycolysis to a relatively less efficient TCA cycle and PPP as sources of energy. In addition, the correlations between various metabolites were also examined through a heatmap of correlation analysis (Fig. S23). Kyoto encyclopedia of genes and genomes (KEGG) enrichment analysis (Fig. 2g) and subsequent pathway analysis (Fig. 2h) revealed glycolysis, the TCA cycle, and the PPP as the most significantly enriched pathways, highlighting the broad impact of glycolysis inhibition on both the classical and bypass glucose metabolic pathways.

Based on previous research, we have unequivocally established glycolysis as a cornerstone of the intricate mechanisms of OS. This pivotal understanding has naturally led us to embark on targeted exploration, centering our primary research endeavors on elucidating the profound effects of SHK@Mn-TiO_2_ on glycolysis. PKM2-specific CLSM images revealed that both the free SHK and SHK@Mn-TiO_2_ nanoplatforms significantly reduced PKM2 expression in K7M2 cells (Fig. 2c). This trend was further accentuated under US irradiation, likely due to increased cell death. Furthermore, western blot (WB) analysis demonstrated these findings, confirming that the downregulation of PKM2 expression was mediated by SHK-based treatments (Fig. 2d). Interestingly, Mn-TiO_2_ alone also exhibited a suppressive effect on PKM2, the downstream protein in the glycolytic pathway (Fig. 2c). Additionally, HK-2, the first rate-limiting enzyme in glycolysis, was also significantly inhibited by both the Mn-TiO_2_ and SHK@Mn-TiO_2_ treatments, as evidenced by the CLSM images (Fig. 2i). These effects were likely complemented by the upregulation of the cGAS-STING pathway, triggered by the extensive release of Mn^2+^ ions [51,52]. Notably, the inhibition of PKM2, a downstream target, appeared to exert a negative feedback effect on HK-2 expression, as evidenced by slightly higher HK-2 levels in the SHK@Mn-TiO_2_ group than in the Mn-TiO_2_ alone group, which was further confirmed by WB analysis (Fig. 2l).

Glycolysis inhibition also led to critical alleviation of the hypoxic TME, thereby enhancing the efficacy of SDT. HIF-1α, a hypoxia-inducible factor typically overexpressed under hypoxic conditions, was significantly downregulated by SHK@Mn-TiO_2_, as demonstrated by CLSM images and WB analysis (Fig. 2k, l) [53]. This downregulation not only provided more O_2_ as a crucial precursor for SDT but also contributed to reversal of the hypoxic TME. Mn-TiO_2_ alone also moderately inhibited HIF-1α expression, which was attributed to its ability to suppress glycolysis via HK-2 downregulation. Upon US irradiation, structural disruption associated with cell death likely led to a more pronounced release of intranuclear HIF-1α. To directly assess the intracellular O_2_ concentration, we utilized the O_2_-sensitive probe [Ru(dpp)_3_]Cl_2_ [54]. CLSM images revealed that [Ru(dpp)_3_]Cl_2_ exhibited red fluorescence, indicative of hypoxia in untreated K7M2 cells. However, pretreatment with SHK and SHK@Mn-TiO_2_ resulted in rapid quenching of this fluorescence due to the presence of O_2_, indicating the reversal of the hypoxic TME (Fig. 2n). Similarly, Mn-TiO_2_ also contributed to increased intracellular O_2_ levels. Finally, the increased SDT induced by SHK@Mn-TiO_2_ was confirmed. By employing the ROS-specific probe 2’,7’-dichlorodihydrofluorescein diacetate (DCFH-DA), we observed an ∼53% increase in ROS generation by SHK@Mn-TiO_2_+US compared with that by Mn-TiO_2_+US, which manifested as intensified green fluorescence in CLSM images and was confirmed by FACS analysis (Figs 2j, m, and S24). Above all, the SHK@Mn-TiO2 nanoplatform achieved dual glycolysis inhibition effects by targeting both PKM2 and HK-2 through SHK and Mn^2+^ ions, respectively. This glycolytic inhibition, coupled with the subsequent increase in the cellular O_2_ concentration, significantly enhanced the efficacy of SDT, which was mediated by the downregulation of the HIF-1α–PKM2 biological axis [55].

### Immunogenic cell death and immune activation elicited by the SHK@Mn-TiO_2_ nanoplatform

**Figure 3.**
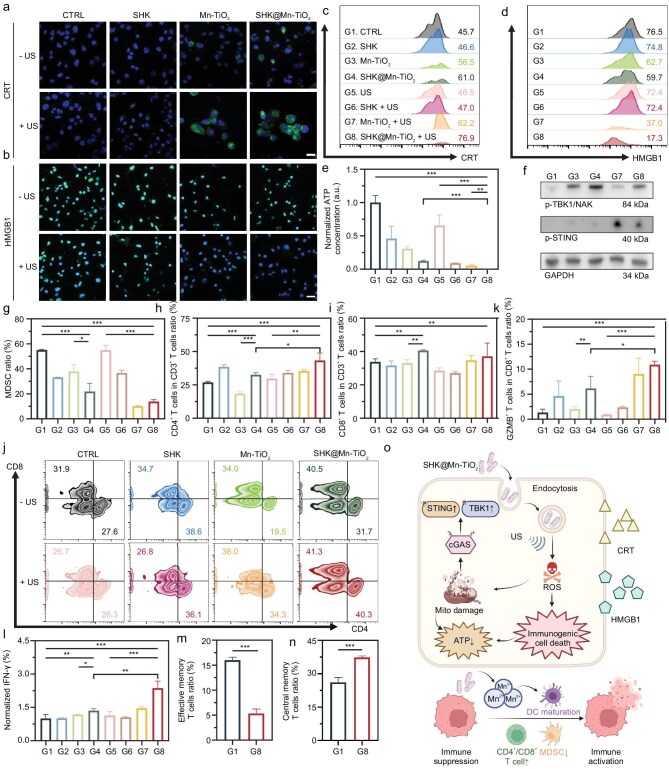
*In vitro* ICD and immune activation induced by SHK@Mn-TiO_2_ under US irradiation*.* CLSM images of CRT- (a) and HMGB1-specific (b) immunofluorescence after different treatments. Scale bar  =  20 μm. FACS analysis of the CRT^+^ (c) and HMGB1^+^ (d) cell ratios after different treatments. Groups 1–8 represent CTRL, SHK, Mn-TiO_2_, SHK@Mn-TiO_2_, US, SHK + US, Mn-TiO_2_ + US, and SHK@Mn-TiO_2_ + US, respectively. (e) Quantitative analysis of ATP concentrations after different treatments. (f) WB analysis of p-TBK1/NAK and p-STING. (g) Quantitative analysis of MDSCs by FACS after different treatments. Quantitative analysis (h, i) and flow dot plots (j) of the ratio of CD4^+^ and CD8^+^ T cells in the CD3^+^ T cell population by FACS after different treatments. (k) Quantitative analysis of the ratio of GZMB^+^ T cells in the CD8^+^ T cell population by FACS after different treatments. (l) Enzyme-linked immunosorbent assay (ELISA) results of IFN-γ in K7M2 cells. Quantitative analysis of CD62L^−^ CD44^+^ Tem (m) and CD62L^+^ CD44^+^ Tcm (n) in T cells co-cultured with treated K7M2 cells. (o) Scheme of ICD and immune activation induced by SHK@Mn-TiO_2_+US *in vitro*. All values are shown as the means  ±  SD (*n*  =  3) *P < 0.05, **P < 0.01, ***P < 0.001.

Cancer immunotherapy has revolutionized cancer treatment strategies through the utilization of advanced biomaterial nanoplatforms, effectively activating immune responses and increasing therapeutic efficacy by activating CTLs [56,57]. This US-activated SDT strategy induces ICD within tumor cells. This process promotes the release of tumor-associated antigens (TAAs) and damage-associated molecular patterns (DAMPs), notably calreticulin (CRT), which functions as an ‘eat me’ signal, and high mobility group protein B1 (HMGB1), which functions as a ‘find me’ signal, thereby igniting a robust immune response against the tumor [58]. Upon exposure to SHK@Mn-TiO_2_ in conjunction with US irradiation, CLSM images and FACS analysis revealed marked upregulation of CRT expression within the cytoplasm and the release of HMGB1 from the nucleus (Figs 3a–d and S25). After SDT treatment, the size of K7M2 cells increased abnormally, indicating structural changes caused by apoptosis resulting from cytotoxicity. These findings reveal the occurrence of ICD and the enhancement of immunotherapy potency. Furthermore, the reduction in ATP levels, a hallmark of ICD, was exacerbated in the SHK@Mn-TiO_2_ group, particularly when combined with US irradiation, severely compromising the energy supply of tumor cells (Fig. 3e).

DCs, vital orchestrators of tumor immunomodulation, were found to be significantly impacted by this treatment. Mn^2+^-mediated activation of the cGAS-STING pathway, coupled with DAMP release from dying cells, promoted DC maturation upon exposure to the nanoplatform [59]. Western blot analysis confirmed the activation of this pathway, as evidenced by the phosphorylation of STING (p-STING) and TBK1/NAK (p-TBK1/NAK), in both the Mn-TiO_2_ and SHK@Mn-TiO_2_ treated groups (Fig. 3f). When supernatants from K7M2 cells treated with Mn-TiO_2_ or SHK@Mn-TiO_2_ were incubated with mouse bone marrow-derived dendritic cells (BMDCs), a notable increase in the expression of the stimulatory markers CD80 and CD86 on DCs was observed, further corroborating their enhanced functionality (Fig. S26). MDSCs, a heterogeneous population of immunosuppressive immature myeloid cells, were also targeted by this treatment. SHK, Mn-TiO_2_, or SHK@Mn-TiO_2_ significantly reduced MDSC induction, with the most pronounced effect observed after SDT. This reduction, though likely influenced by the inherent fragility of MDSCs, underscored the immunomodulatory effects of the treatment and highlighted the need for further *in vivo* validation (Figs 3g and S27).

Importantly, decreasing MDSC numbers can increase the survival of CTLs, particularly CD8^+^ T cells, which are pivotal in tumor eradication [60]. T cells derived from mouse spleens, including CD3^+^ CD4^+^, CD3^+^ CD8^+^, and granzyme B^+^ (GZMB^+^) CD8^+^ T cells, significantly increased after incubation with SHK@Mn-TiO_2_ in conditioned medium, indicating the activation of CTLs and the subsequent potentiation of immunotherapy against K7M2 tumors (Figs 3h–k, S28, and S29). Moreover, the levels of IFN-γ were significantly elevated in tumor tissues after SHK@Mn-TiO_2_+US treatment (Fig. 3l). Effective memory T cells (Tem) and central memory T cells (Tcm) were considered as the most important memory T cells and could induce strong immune memory effects. FACS analysis confirmed a significant increase in the proportions of Tem and a decrease in the ratio of Tcm after being co-cultured with the K7M2 cells of the SHK@Mn-TiO_2_+US group, indicating a rapid shift from Tcm to Tem cells to induce the immune memory (Figs 3m, n, and S30) [61]. Overall, the ROS generated by SHK@Mn-TiO_2_ combined with US irradiation elicited profound ICD effects in OS cells. The dual inhibition of glycolysis further disrupted ATP production, effectively starving tumor cells. Notably, the combined effects of Mn^2+^-mediated cGAS-STING pathway activation and glycolysis inhibition transformed the typically low-immunogenic ‘cold’ tumor microenvironment of K7M2 into a highly immunogenic ‘hot’ tumor, thereby enhancing the overall immune response and therapeutic outcome (Fig. 3o).

### SHK@Mn-TiO_2_-based enhanced SDT for OS treatment and glycolysis inhibition *in vivo*

**Figure 4.**
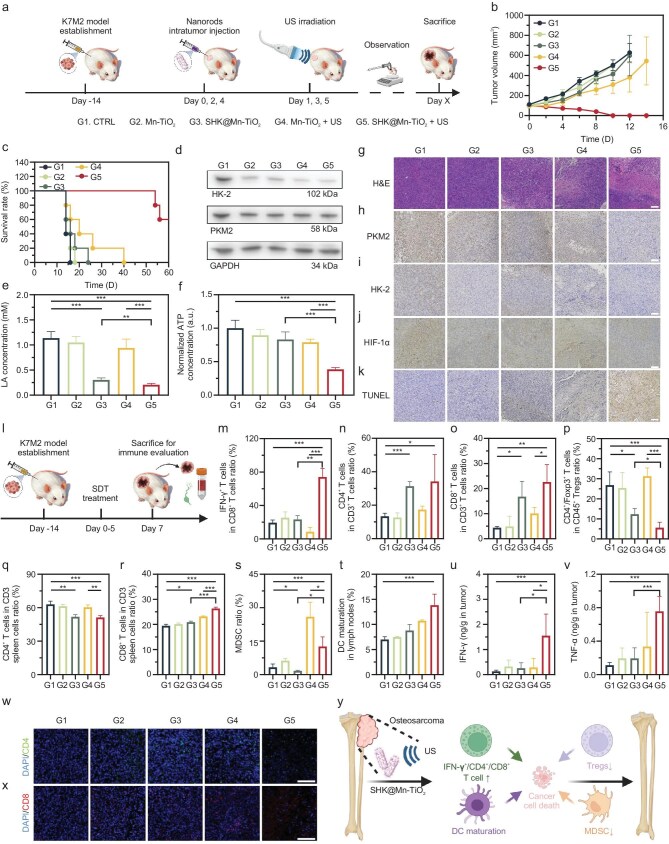
*In vivo* therapeutic outcomes of the enhanced sono-immune strategy based on SHK@Mn-TiO_2_+US in the K7M2 model. (a) Schematic illustration and treatment timeline of the glycolysis inhibition enhanced sono-immune strategy in the K7M2 tumor model. Groups 1–5 represent CTRL, Mn-TiO_2_, SHK@Mn-TiO_2_, Mn-TiO_2_ + US, and SHK@Mn-TiO_2_ + US. Average tumor volume curves (b) and overall survival rates (c) after different treatments. (d) WB analysis of HK-2 and PKM2 expression in the living tumors on day 7 post treatment. Quantitative analysis of LA (e) and ATP concentrations (f) in living tumors on day 7 post treatment. (g) Representative H&E staining of tumor sections from living tumor on day 7 post treatment. Representative immunohistochemical staining of tumor sections for PKM2 (h), HK-2 (i), HIF-1α (j), and TUNEL (k) in living tumors on day 7 post treatment. Scale bar  =  100 μm. (l) Schematic illustration and immune evaluation timeline of the glycolysis inhibition enhanced sono-immune strategy in the K7M2 tumor model. Quantitative analysis of IFN-γ^+^ CD8^+^ T cells in tumor (m), CD3^+^ CD4^+^ T cells in tumor (n), CD3^+^ CD8^+^ T cells in tumor (o), CD4^+^ Foxp3^+^ Tregs in tumor (p), CD3^+^ CD4^+^/CD4^+^ CD8^+^ T cells in spleen (q, r), CD11b^+^ Gr-1^+^ MDSCs in tumor (s), and CD80^+^ CD86^+^ matured DCs in TDNLs (t) by FACS analysis in the living tumors on 7 days post-treatment. ELISA results of IFN-γ (u) and TNF-α (v) in tumors. Representative CLSM images of CD4^+^ (w) and CD8^+^ T cells (x) in tumors measured by immunofluorescent staining. Scale bar  =  100 μm. (y) Scheme of the immune evaluation of the glycolysis inhibition enhanced sono-immune strategy based on SHK@Mn-TiO_2_+US in the K7M2 model *in vivo*. All values are shown as the means  ±  SD (*n*  =  5) *P < 0.05, **P < 0.01, ***P < 0.001.

The remarkable sonodynamic and immune-modulatory properties of SHK@Mn-TiO_2_ prompted an in-depth evaluation of its antitumor efficacy on Balb/c mice bearing subcutaneous K7M2 tumors. Initially, a double therapeutic dose of SHK@Mn-TiO_2_ (10 mg/kg) was subcutaneously injected into healthy mice to assess its biocompatibility *in vivo*. After a 30-day observation period, hematoxylin and eosin (H&E) staining of major organs, including the heart, liver, spleen, lungs, kidneys, and skin, revealed no discernible organ damage (Fig. S31). In addition, complete blood count and serum biochemical analysis confirmed the outstanding biosafety of SHK@Mn-TiO_2_ even at high doses, supporting the feasibility of the sono-immune strategy (Fig. S32). Systemic administration of high dose SHK@Mn-TiO_2_ for 60 days resulted in minor organ damage and hemogram change, further supporting its application *in vivo* (Figs S33 and S34). Balb/c mice bearing subcutaneous K7M2 tumors were randomly assigned to five treatment groups (Fig. 4a): (1) control group, (2) Mn-TiO_2_ (5 mg/kg) group, (3) SHK@Mn-TiO_2_ (5 mg/kg) group, (4) Mn-TiO_2_ (5 mg/kg) + US (30 kHz, 10 W/cm^2^, 10 min) group, and (5) SHK@Mn-TiO_2_ (5 mg/kg) + US (30 kHz, 10 W/cm^2^, 10 min) group. As expected, the SHK@Mn-TiO_2_+US group exhibited a profound antitumor response with ∼60% survival rate in the long term, which was characterized by significant tumor growth inhibition and prolonged survival, approaching the efficacy of radical tumor treatments (Fig. 4b, c). The detailed tumor volumes of individual mice in the SHK@Mn-TiO_2_+US group continuously decreased over the first 10 days, but tumor recurrence occurred in ∼40% of them at ∼38 days (Fig. S35). While Mn-TiO_2_ combined with US irradiation demonstrated modest antitumor effects, the long-term outcomes were suboptimal, similar to those of the single SHK@Mn-TiO_2_ group. Throughout the study, the stable body weights of the mice across all groups further validated the biocompatibility of these treatments (Fig. S36).

To delve into the modulation of glycolysis within the K7M2 TME, tumors were excised on day 7 for analysis. Notably, the protein expression levels of HK-2 and PKM2 were significantly reduced in the treatment groups, which was attributed to the synergistic effects of Mn^2+^ and SHK (Fig. 4d). Additionally, SHK@Mn-TiO_2_ markedly decreased LA production (Fig. 4e). Although Mn-TiO_2_ alone or combined with US irradiation also resulted in a reduction in the LA concentration, the effect was not statistically significant, indicating the metabolic resistance of tumor cells. In K7M2 cells, a single Mn^2+^ cannot break through such negative feedback of cell self-protection, emphasizing the importance of combined therapy. The intracellular ATP levels in K7M2 tumors, which were intimately linked to glycolysis, tended to decrease in the SHK@Mn-TiO_2_ group, with an even more pronounced decrease upon combination with US. This ATP reduction was attributed to the combined influence of SHK-mediated glycolysis inhibition and SDT-induced ICD, in contrast to the effects of Mn-TiO_2_ and Mn-TiO_2_+US alone (Fig. 4f).

To further validate the therapeutic outcomes, H&E staining was performed on tumors from all the treatment groups 7 days post-treatment. The SHK@Mn-TiO_2_+US group, where glycolysis inhibition was coupled with SDT, exhibited pronounced cell death (Fig. 4g). Conversely, no significant antitumor effects were observed in the control or Mn-TiO_2_ groups. Immunohistochemical staining revealed a marked decrease in the protein expression of PKM2, HK-2, and HIF-1α following SHK@Mn-TiO_2_+US treatment, indicating the reversal of glycolysis and the hypoxic TME (Fig. 4h–j). Furthermore, terminal deoxynucleotidyl transferase dUTP nick-end labelling (TUNEL) staining confirmed that the highest level of tumor apoptosis occurred in the SHK@Mn-TiO_2_+US group (Fig. 4k). Notably, the level of apoptosis in the Mn-TiO_2_+US group was greater than that in the SHK@Mn-TiO_2_ group alone, highlighting SDT as the primary inducer of tumor apoptosis. Therefore, the potent antitumor efficacy of SHK@Mn-TiO_2_ in combination with US irradiation has been well demonstrated in treatment of OS in mouse models. This nanoplatform exhibited exceptional biocompatibility and safety profiles, even at elevated doses. The synergistic effects of glycolysis inhibition and SDT-induced ICD likely contributed to this efficacy.

### SHK@Mn-TiO_2_ SDT for TME modulation in OS treatment

The therapeutic potential of SHK@Mn-TiO_2_-based SDT in modulating the TME for OS treatment was profoundly examined. One week after treatment, comprehensive immune evaluations were conducted on harvested tumors, tumor-draining lymph nodes (TDLNs), spleens, and blood samples from K7M2 tumor-bearing mice (Fig. 4l). CD8^+^ T cells have been shown to be closely associated with a good prognosis in tumor patients [62]. Encouragingly, the SHK@Mn-TiO_2_+US treatment group presented a substantial increase in the proportions of vital T helper cells (Th cells, 157% increase in CD3^+^ CD4^+^) and cytotoxic T lymphocytes (CTLs, 280% increase in IFN-γ^+^ CD8^+^ and 35% increase in CD3^+^ CD8^+^) compared with those of the control groups, indicating a robust immunotherapeutic response against K7M2 tumors (Figs 4m–o and S37a–S37c). A significant decrease in CTLs in the Mn-TiO_2_+US group was observed, leading to unsatisfactory immune function. The incorporation of SHK notably reduced the ratio of immunosuppressive CD4^+^ Foxp3^+^ Tregs in both the SHK@Mn-TiO_2_ and SHK@Mn-TiO_2_+US groups, highlighting the substantial role of glycolysis inhibition in reversing immune suppression (Figs 4p and S37d). However, the proportion of CD3^+^ CD4^+^ T cells in the spleen decreased, possibly attributed to their migration toward the tumor site (Figs 4q and S37e). Moreover, the number of CD3^+^ CD8^+^ T cells in the spleen showed a similar increasing trend to that in the tumor, amplifying immune-mediated tumor destruction (Figs 4r and S37e). A similar phenomenon was reported in a previous study. CD8^+^ T cells expanded in both lymphoid and peripheral organs, whereas CD4^+^ T cells showed no significant expansion in either, caused by the phenotype variety of these expanded cells in peripheral organs [63]. In terms of the total amount, the spleen and tumor-infiltrating CD4^+^ T cells were significantly more than CD8^+^ T cells, mainly because of the higher output of CD4^+^ T cells in thymus [64]. In addition, previous studies indicated that CD8^+^ T cells in peripheral immune organs have higher proliferation activity and turnover rate, compared with CD4^+^ T cells, leading to the content differences of CD4^+^ and CD8^+^ T cells in the spleen and tumor immune microenvironment [65,66].

A detailed analysis of myeloid-derived suppressor cells (MDSCs) revealed an increase in their proportion in the Mn-TiO_2_ group, whereas their numbers were markedly reduced below control levels in the SHK@Mn-TiO_2_ group, suggesting the inhibitory effects of SHK on MDSCs (Figs 4s and S37f). Notably, under the SDT effects of Mn-TiO_2_+US, the number of MDSCs dramatically increased to 25.9%, exacerbating immunosuppression. The MDSC ratio of the SHK@Mn-TiO_2_+US group was less than half that of the Mn-TiO_2_+US group, owing to the glycolysis-inhibiting properties of SHK. Consistent with the *in vitro* findings, all the treatment groups presented varying degrees of DC maturation, triggered by the release of Mn^2+^ and tumor debris during SDT (Figs 4t and S37g). Additionally, tumor-associated macrophages (TAMs) were polarized toward the pro-inflammatory M1 phenotype by ROS at the tumor site, fostering adaptive immunity in conjunction with mature DCs (Fig. S38a–S38d). Moreover, the levels of TNF-α and IFN-γ were significantly elevated in tumor tissues after SHK@Mn-TiO_2_+US treatment (Fig. 4u, v). TDLNs play a critical role in immune memory [67]. A similar upward trend was observed in TDLNs and blood, suggesting the activation of a prolonged immune memory effect (Fig. S39). Immunofluorescence staining further corroborated these findings, showing intensified green fluorescence corresponding to CD4 and CD8, indicative of enhanced tumor immune activation mediated by SHK@Mn-TiO_2_+US (Fig. 4w, x). Overall, the intratumoral injection of SHK@Mn-TiO_2_ combined with US irradiation elicited ROS-mediated cytotoxic effects via SDT on K7M2 tumors. This led to the release of tumor debris, which profoundly promoted DC maturation, triggering a cascade of tumor-specific immune responses. These responses were characterized by increased infiltration of Th cells and CTLs within the tumor, contributing to immune-mediated tumor eradication in OS. Concurrently, glycolysis inhibition by SHK mitigated immune suppression, as evidenced by the reduced proportions of Tregs and MDSCs, thereby increasing the efficacy of immunotherapy (Fig. 4y).

### SHK@Mn-TiO_2_ based SDT results in sustained immune memory in OS

**Figure 5.**
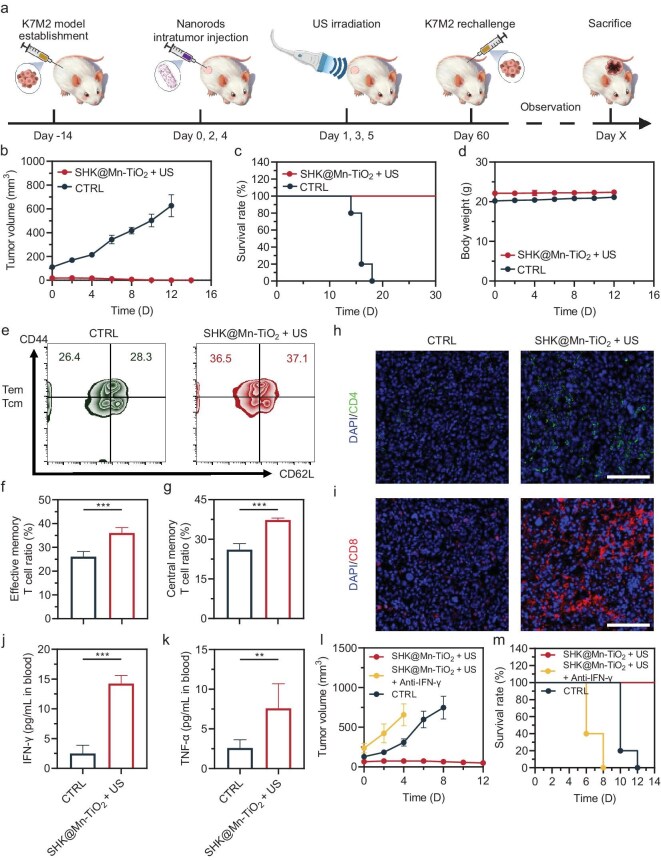
Therapeutic outcomes and immune evaluation of the glycolysis inhibition enhanced sono-immune strategy. (a) Schematic illustration and timeline of the glycolysis inhibition enhanced sono-immune strategy in the K7M2 rechallenge model. Average tumor volume curves (b), overall survival rates (c), and body weights (d) after different treatments in rechallenge models. (e) Flow dot plots of CD62L^−^ CD44^+^ Tem and CD62L^+^ CD44^+^ Tcm in the living tumor at 7 days post-rechallenge. Quantitative analysis of CD62L^−^ CD44^+^ Tem (f) and CD62L^+^ CD44^+^ Tcm (g) in the living tumors at 7 days post-rechallenge. Representative CLSM images of CD4^+^ (h) and CD8^+^ T cells (i) in tumors measured by immunofluorescence staining. Scale bar  =  100 μm. ELISA results of IFN-γ (j) and TNF-α (k) in the blood 60 days after primary treatment. Average tumor volume curves (l) and overall survival rates (m) after different treatments in rechallenge models. All values are shown as means  ±  SD (*n*  =  5) *P < 0.05, **P < 0.01, ***P < 0.001.

Encouraged by the robust immune responses induced by the synergistic action of SDT and glycolysis inhibition, we investigated the potential of SHK@Mn-TiO_2_ for stimulating sustained immune memory. By utilizing the potent therapeutic ability of SHK@Mn-TiO_2_+US, we subjected the surviving K7M2 tumor-bearing mice to rechallenge with K7M2 cells via subcutaneous injection on the contralateral back precisely 60 days after the primary treatment (Fig. 5a). For comparative purposes, the healthy mice were also inoculated with tumor cells, and their tumor volumes and body weights were meticulously monitored over time. Strikingly, the rechallenge outcomes for the SHK@Mn-TiO_2_+US group exhibited a remarkable ability to suppress tumor growth, with minimal proliferation observed during the initial logarithmic phase, followed by a nearly complete cessation of further growth (Figs 5b and S40). Throughout the entire observation period, the treated mice maintained a 100% survival rate and stable body weights, confirming the exceptional immune memory functionality and biocompatibility inherent to SHK@Mn-TiO_2_+US (Fig. 5c, d). Furthermore, FACS analysis confirmed a significant increase in the proportions of both Tem and Tcm, two pivotal components of immune memory, further confirming the activation of immune memory by SHK@Mn-TiO_2_+US (Fig. 5e–g). Immunofluorescence staining of rechallenged tumor tissues revealed increased infiltration of CD4^+^ and CD8^+^ T cells in the SHK@Mn-TiO_2_+US group, providing compelling evidence of the long-lasting and robust immune memory activation achieved by this innovative therapeutic approach (Fig. 5h, i). On the day of K7M2 rechallenge, blood analysis revealed substantial increases in the levels of TNF-α and IFN-γ, suggesting the robust presence of circulating immune memory even 60 days after initial treatment (Fig. 5j, k). Interestingly, IFN-γ seemed to play an important role in anti-tumor immune response induced by SHK@Mn-TiO_2_+US, based on the boosting of IFN-γ^+^ T cells and increased IFN-γ in circulation. As a result, the tumor volumes of K7M2 rechallenge models in the anti-IFN-γ group significantly increased, even exceeding that of the control group, indicating the impact on IFN-γ in immune memory in OS (Figs 5l, m, and S41) [68]. These findings collectively underscored the immense potential of SHK@Mn-TiO_2_-based SDT, which was augmented by glycolysis inhibition, as a promising strategy for the treatment of OS and was capable of fostering durable immune memory against recurrent disease.

## CONCLUSION

In summary, this comprehensive study demonstrated the potent antitumor immunotherapeutic effects of SHK@Mn-TiO_2_ combined with US irradiation. The bioinformatic analysis of clinical OS samples revealed elevated glycolysis and upregulated expression of the pivotal enzyme PKM2, which was subsequently corroborated at the cellular level. The introduction of SHK@Mn-TiO_2_ effectively inhibited PKM2 expression, while the released Mn^2+^ ions ignited the cGAS-STING pathway and concurrently downregulated HK-2, another vital glycolytic enzyme, thereby executing a dual-pronged inhibition of glycolysis. Notably, the integration of SHK diminished intracellular oxygen consumption and drastically reduced HIF-1α levels, reversing the hypoxic TME and substantially decreasing lactate accumulation. In this oxygen-replenished TME, SHK@Mn-TiO_2_ energized by US irradiation exhibited increased sonodynamic therapy efficacy and increased tumor cytotoxicity.

This innovative sono-immune approach, which synergistically integrated glycolysis inhibition with SDT, demonstrated remarkable therapeutic outcomes and immune activation in a K7M2 tumor model. Specifically, SHK@Mn-TiO_2_+US successfully counteracted the immunosuppressive TME typically induced by conventional SDT, manifesting as elevated extracellular levels of IFN-γ and TNF-α. These cytokines, in turn, enhanced CTL infiltration and DC maturation, while concurrently diminishing the proportions of immunosuppressive Tregs and MDSCs. Furthermore, we revealed robust immune memory effects in a K7M2 tumor rechallenge model, highlighting the long-term protective immunity elicited by this treatment strategy. This research introduced a novel sono-immune strategy that combines glycolysis inhibition with SDT, elucidated the intricate synergistic mechanisms underpinning its efficacy, and presented a promising avenue for addressing the current therapeutic challenges in OS treatment.

In the clinic, US is one of the most commonly used imaging techniques due to its excellent biological effects. SDT, as an alternative approach for tumor treatment mediated by US, has been investigated in clinical practice and seems to be a promising strategy [23,69]. In addition, researchers are striving to increase SDT efficiency by constructing multifunctional nanoplatforms that can regulate the TME by ROS production, immune activation, and immunosuppression reversion [70]. Despite the rapid development of combined sono-immune strategy, several challenges, such as curative effect, specificity, and long-term safety, remains to be overcome for further use in clinical cancer treatment [71].

## METHODS

### Statistical analysis

Data are presented as means ± SD. Statistical significance was determined using the appropriate method based on the distribution of the data. For normally distributed numerical data, statistical significance was calculated using Student's *t*-test. In cases where multiple groups were compared and data were normally distributed with equal variances, one-way ANOVA (Analysis of Variance) was employed, followed by *post-hoc* tests for pairwise comparisons if significant differences were detected. For non-normally distributed data, Kruskal–Wallis was employed as the non-parametric alternative. Survival analysis was calculated by the Log-rank test. The significance is expressed as ns *p* > 0.05, *p < 0.05, **p < 0.01, and ***p < 0.001.

## Supplementary Material

nwaf365_Supplemental_File
