# Supplementary material for: Sonodynamic therapy augmented by glycolysis inhibition: a novel metabolic reprogramming strategy for enhanced osteosarcoma treatment
Source: Natl Sci Rev. 2025 Aug 30;12(11):nwaf365. doi: 10.1093/nsr/nwaf365 (PMC12573261; doi:10.1093/nsr/nwaf365)
Supplement: nwaf365_Supplemental_File [file nwaf365_supplemental_file.pdf]

Supporting Information

Sonodynamic Therapy Augmented by Glycolysis Inhibition: A Novel Metabolic Reprogramming Strategy for Enhanced Osteosarcoma Treatment

Zhuorun Song[1,2,#], Shunyi Lu[1,2,#], Yuqi Yang[2], Zijun Chen[3], Youdong Chen[2], Jie Cao[2], Zimin Zhang[1], Jun Ge[1,2,*], Huilin Yang[1,*] and Liang Cheng[2,*]

1. Department of Orthopedic Surgery, The First Affiliated Hospital of Soochow University, Suzhou, 215006, China

2. Institute of Functional Nano & Soft Materials (FUNSOM), Jiangsu Key Laboratory for Carbon-Based Functional Materials & Devices, Soochow University, Suzhou, 215123, China

3. Department of Breast Surgery, Obstetrics & Gynecology Hospital of Fudan University， Yangtze River Delta Integration Demonstration Zone (Qingpu), Shanghai, 201799, China

#These authors contributed equally to this work.

*Corresponding authors: lcheng2@suda.edu.cn; hlyang@suda.edu.cn; gejun115@suda.edu.cn

1. Experiment and Methods

Chemicals

All chemicals used in the experiments were purchased from Sigma-Aldrich unless otherwise described. Commercially available reagents were used without further purification unless noted otherwise. The primary antibodies and secondary antibodies were purchased from Abcam. All the flow cytometry antibodies were purchased from Biolegend.

Characterization

The morphology of SHK@Mn-TiO_2 was characterized by the transmission electron microscopy (TEM, FEI TF20, Tecnai). The crystal structure and surface chemical composition of SHK@Mn-TiO_2 were measured by X-ray diffraction (XRD, Panalytical Empyrean) and X-ray photoelectron spectroscopy (XPS, ESCALab 250Xi). The hydrodynamic size and zeta potential were recorded by a Malvern Zeta sizer (Nano ZS90, Malvern). The absorption spectra were obtained by a UV-vis-NIR spectrophotometer (Thermo50 UV-vis-NIR spectrophotometer, Thermo Scientific). The absolute concentration of Mn ions was measured via an inductively coupled plasma optical emission spectrometer (ICP-OES, Avio 200, PerkinElmer). The flow cytometry was performed with a flow cytometer (C6 plus, BD). The cell fluorescence images were observed via the confocal laser scanning microscopy (CLSM, LSM 800, Zeiss). Ultrasound (US) irradiation was performed by the focused ultrasound system (Nasonic).

Data collection and scRNA-Seq data analysis

The gene expression matrix of GSE99671 was downloaded from GEO. The expression level of PKM2 was subsequently visualized using boxplot via ggplot2, and differential analysis between normal and tumor samples in GSE99671 was performed using DESeq2. The difference analysis results were visualized using ggplot2. All tumor samples in GSE99671 were grouped according to the median expression of PKM2 (low, high). xCell was used to perform immune infiltration analysis on all samples in GSE99671, and immune cell scores were obtained. The immune cell scores in the PKM2 low vs high groups were visualized by ggplot2. Immune cells with significantly different immune cell scores in the PKM2 low vs high groups were extracted respectively. Spearman was used to calculate the correlation and a corrplot was used to plot the correlation heat map.

Celluar lactate assay

For analysis of lactate production between normal cells and tumor cells, sorted cells (1×10^6) including Raw 264.7 cells, K7M2 cells, HUVEC cells, and MG-63 cells, were collected, and then, the cell extraction solutions were obtained and detected via a lactate assay kit according to the manufacturer instructions.

Synthesis of Mn-TiO_2

Taking $TiCl_4$ as a precursor, Mn-TiO_2 was fabricated via a kind of high-temperature organic-phase approach. Specifically, 220 μL of $TiCl_4$ (2 mmol) was dispersed in 20 mL of OM and 15 mL of ODE in a three-necked flask under magnetic stirring. This mixture was heated to 140 ℃ and stirred

for 20 mins under a N_2 atmosphere to remove the water and oxygen. Then, this mixture was heated to 260 ℃ at this temperature for 20 mins. Next, a S/OM solution, prepared by dissolving 128 mg of S powder (4 mmol) in 5 mL of OM, was injected into the flask at 260 ℃ for 20 mins. Then, the mixture was heated to 300 ℃ at this temperature for 20 mins. After being cooled down to room temperature, the Mn-TiO_2 samples were washed with cyclohexane and ethanol for three times.

Modification of SHK@Mn-TiO_2

To enhance the biocompatibility, 1,2-distearoyl-sn-glycero-3-phosphoethanolamine modified polyethylene glycol (MW = 2000, DSPE-PEG2000) and shikonin (SHK) were employed to modify Mn-TiO_2. Speifically, 50 mg of DSPE-PEG2000 and 0.2 mg of SHK was dissolved in 4 mL of chloroform, followed by the addition of 10 mg of Mn-TiO_2 dispersed in 1 mL of chloroform under sonication for 10 mins. N_2 was used to dry the solution to obtain the final solid sample SHK@Mn-TiO_2, which was centrifugated at 3000 rpm for 5 min, dispersed in deionized water and stored at 4 ℃ with N_2 protection for future use.

Determination of US parameters

To determine the optimum US, K7M2 cells were subjected to different parameters (3 W/cm^2 for 3 min, 3 W/cm^2 for 5 min, 5 W/cm^2 for 3 min, 5 W/cm^2 for 5 min, 10 W/cm^2 for 3 min, and 10 W/cm^2 for 5 min). A standard 3-(4,5-dimethyl-2-thiazolyl)-2,5-diphenyl-2-htetrazolium bromide (MTT) assay was performed to determine the relative cell viability (n = 3).

Sonodynamic effect of SHK@Mn-TiO_2

To detect the $^1O_2$ generation of SHK@Mn-TiO_2 via sonodynamic effects, SHK@Mn-TiO_2 (10 μg/mL) was mixed with 40 μL of 1,3-diphenylisobenzofuran (DPBF, 1 mg/mL in ethanol) under ultrasound (US) irradiation (30 kHz, 3 W/cm^2). After US irradiation for different time and treatment, the decrease in the absorbance of DPBF at 420 nm determined the generation of $^1O_2$.

For ESR measurements, $^1O_2$ was detected by a 2,2,6,6-tetramethylpiperidine (TEMP) probe. SHK@Mn-TiO_2 (10 μg/mL) was mixed with 20 μL of TEMP and exposed to US irradiation (30 kHz, 3 W/cm^2) for 3 min. The characteristic peak signals were detected via an electron spin resonance (ESR) spectrometer.

Fenton-like reaction of SHK@Mn-TiO_2

To detect the ROS generated by the reaction with H_2O_2, 10 μL of TMB (20 mg/mL in dimethyl sulfoxide) was added into SHK@Mn-TiO_2 (10 μg/mL) containing 100 μM H_2O_2. After the reaction for different time, the change in the absorbance of TMB at 662 nm reflected the generation of ROS by the Fenton-like reaction of SHK@Mn-TiO_2.

Mn^{2+} release of SHK@Mn-TiO_2

To detect Mn^{2+} release from SHK@Mn-TiO_2, SHK@Mn-TiO_2 (1 mg/mL) was added into a dialysis bag (MWCO = 5000 Da). After stirring in double distilled water for different time, the supernatants

were collected and further dealt with aqua regia at 300 ℃ for 5 min. The Mn^{2+} concentrations of these supernatants were measured via ICP-OES.

Stability of SHK@Mn-TiO_2

To confirm the stability of SHK@Mn-TiO_2, SHK@Mn-TiO_2 (100 μg/mL) was dissolved in H_2O or DMEM containing 10% FBS, respectively. The absorbance of both solutions on days 1, day 3, day 5, and day 7 were recorded by UV-vis-NIR spectrophotometer. Images on days 1 and 7 were also captured.

Cell culture

Human umbilical vein endothelial cells (HUVECs), the human cervical carcinoma cell line Hela, the murine osteosarcoma cell line K7M2, and the murine macrophage line RAW 264.7 were obtained from the American Type Culture Collection (ATCC) and cultured in complete DMEM in a standard cell culture environment (37 ℃, 5% CO_2). Human osteosarcoma cell line MG-63 was obtained from the American Type Culture Collection (ATCC) and cultured in complete DMEM with 10% Fetal bovine serum (FBS, F101, Vazyme Biotech Co. Ltd) under a standard cell culture environment (37 ℃, 5% CO_2). All cells were collected from the culture dishes by trypsase (Dakewe).

Cellular experiments

The treatment groups used in the cellular experiments described below included the CTRL, SHK (1 μg/mL), Mn-TiO_2 (50 μg/mL), SHK@Mn-TiO_2 (50 μg/mL), US, SHK (1 μg/mL) + US, Mn-TiO_2 (50 μg/mL) + US, and SHK@Mn-TiO_2 (50 μg/mL) + US.

To assess cell toxicity of SHK@Mn-TiO_2, different concentrations of SHK@Mn-TiO_2 (0, 1, 2, 5, 10, 20, 50, and 100 μg/mL) were incubated with RAW 264.7. The standard MTT assay was performed to determine the relative cell viability after 24 h (n = 3).

For the cellular uptake of SHK@Mn-TiO_2, K7M2 cells were incubated with Cy5.5-labeled SHK@Mn-TiO_2 (50 μg/mL) for 12 h. After washing with Phosphate Buffered Saline (PBS) three times, the collected cells were blocked with 4% paraformaldehyde for 30 mins. All the cell fluorescence images were captured via CLSM.

For *in vitro* cell viability, K7M2 cells were incubated with different treatments for 12 h. Then the groups treated with US were further incubated for 3 h after US (30 kHz, 3 W/cm^2, 3 min). An MTT assay was performed to determine the relative cell viability after different treatments for 24 h (n = 3).

To detect the intracellular O_2 concentration, K7M2 cells were first cultured with an anaerobic gas-producing bag for 12 h, followed by incubation with tris (4,7-diphenyl-1,10-phenanthroline) ruthenium (II) dichloride complex $[Ru(dpp)_3]Cl_2$ (20 μg/mL) for another 3 h. Then, different treatments were conducted for 12 h. After washing with PBS three times, the collected cells were

blocked with 4% paraformaldehyde for 30 mins. All the cell fluorescence images were captured via CLSM.

To detect cellular ROS generation, K7M2 cells were incubated with different treatments for 12 h. Then the groups treated with US were further incubated for 3 h after US (30 kHz, 3 W/cm^2, 3 min). For flow cytometry, the collected cells were first blocked with 5% FBS. After washing with PBS three times, the collected cells were incubated with dichlorodihydrofluorescein diacetate (DCFH-DA, 20 μM) for 1 h to capture intracellular ROS and tested by a C6 plus (n = 3). For CLSM, the collected cells were blocked with 5% FBS. After washing with PBS three times, the collected cells were incubated with DCFH-DA (20 μM) for 1 h to capture intracellular ROS. All the cell fluorescence images were acquired via CLSM.

To evaluate mitochondrial oxidative phosphorylation, K7M2 cells were incubated with different treatments for 12 h. Then the groups treated with US were further incubated for 3 h after US (30 kHz, 3 W/cm^2, 3 min). The $NADP^+$/NADPH ratio assay was conducted according to the recommended protocol. The absorbance of the samples above at 420 nm was used to determine the degree of mitochondrial oxidative phosphorylation.

To measure the mitochondrial membrane potential, K7M2 cells were incubated with different treatments for 12 h. Then, the groups treated with US were further incubated for 3 h after US (30 kHz, 3 W/cm^2, 3 min), followed by staining with the lipophilic cationic probe

5,5',6,6'-tetrachloro-1,1',3,3'-tetraethyl-imida-carbocyanine iodide (JC-1) for 1 h according to the recommended protocol. The collected cells were washed and tested with a flow cytometer (n = 3).

For live/dead dual-staining *in vitro*, K7M2 cells were incubated with different treatments for 12 h. Then, the groups treated with US were further incubated for 3 h after US (30 kHz, 3 W/cm^2), followed by staining with calcein AM and propidium iodide (PI) for 1 h according to the recommended protocol. All the cell fluorescence images were acquired by CLSM.

To verify the relative protein expression *in vitro*, K7M2 cells were incubated with different treatments for 12 h. Then, the groups treated with US were further incubated for 3 h after US (30 kHz, 3 W/cm^2, 3 min), after which they were collected and suspended in 4 ℃ cell lysis buffer. The protein quantification was conducted by BCA protein content assay kit (AKPR017, Beijing Boxbio Science & Technology Co.,Ltd.) before western blotting in the ExpressPlus™ PAGE Gels (12%, GenScript) according to the recommended protocol with prestained protein marker (MR-WB-02, Ketu Biotech).

To test the expression of PKM2 and HK-2, K7M2 cells were incubated with different treatments for 12 h. Then, the groups treated with US were further incubated for 3 h after US (30 kHz, 3 W/cm^2, 3 min). The collected cells were first blocked with 5% FBS at room temperature and then incubated with primary antibody diluted by the universal antibody diluent (WB100D, New Cell & Molecular Biotech) for 12 h at 4 ℃. After being washed with PBS three times, the cells were then incubated

with secondary antibody (Alexa Fluor 488, Abcam, USA) for 1 h at room temperature according to the recommended protocol. All the cell fluorescence images were acquired by CLSM.

To test the expression of HIF-1α, K7M2 cells were incubated with different treatments for 12 h. Then the groups treated with US were further incubated for 3 h after US (30 kHz, 3 W/cm^2, 3 min). The collected cells were first blocked with 5% FBS and 0.1% Triton-100 at room temperature and then incubated with primary antibody and 0.1% Triton-100 for 12 h at 4 ℃. After being washed with PBS three times, the cells were then incubated with secondary antibody and 0.1% Triton-100 for 1 h at room temperature according to the recommended protocol. All the cell fluorescence images were acquired by CLSM.

To test CRT expression, K7M2 cells were incubated with different treatments for 12 h. Then the groups treated with US were further incubated for 3 h after US (30 kHz, 3 W/cm^2, 3 min). For flow cytometry, the collected cells were first blocked with 5% FBS at room temperature and incubated with a primary antibody for 12 h at 4 ℃. After being washed with PBS three times, the cells were then incubated with Goat Anti-Rabbit IgG/SAlexa Fluor 488 (Beijing Solarbio Science & Technology Co., Ltd.) for 1 h at room temperature according to the recommended protocol. The collected cells were washed with PBS and tested by C6 plus (n = 3). For CLSM, the collected cells were blocked with 5% FBS. The cells were incubated with primary antibody for 12 h at 4 ℃. After being washed with PBS three times, the cells were then incubated with secondary antibody for 1 h at room temperature according to the recommended protocol. All the cell fluorescence images were acquired by CLSM.

To test HMGB1 expression, K7M2 cells were incubated with different treatments for 12 h. Then the groups treated with US were further incubated for 3 h after US (30 kHz, 3 W/cm^2, 3 min). For flow cytometry, the collected cells were first blocked with 5% FBS and 0.1% Triton-100 at room temperature and then incubated with a primary antibody supplemented with 0.1% Triton-100 for 12 h at 4 ℃. After being washed with PBS three times, the cells were then incubated with secondary antibody for 1 h with 0.1% Triton-100 at room temperature according to the recommended protocol. The collected cells were washed with PBS and tested by C6 plus (n = 3). For CLSM, the collected cells were blocked with 5% FBS and 0.1% Triton-100. The cells were incubated with primary antibody with 0.1% Triton-100 for 12 h at 4 ℃. After being washed with PBS three times, the cells were then incubated with secondary antibody and 0.1% Triton-100 for 1 h at room temperature according to the recommended protocol. All the cell fluorescence images were acquired by CLSM.

To measure ATP release, K7M2 cells were incubated with different treatments for 12 h. Then the groups treated with US were further incubated for 3 h after US (30 kHz, 3W/cm^2, 3 min). The cells were lysed under ultrasound (with a power of 25%, sonication for 3 s, and pause for 10 s, this process was repeated 30 times) on ice and centrifuged to collect the supernatants. The collected supernatants were measured with an ATP Assay Kit according to the recommended protocol (n = 3).

To promote DCs maturation, bone marrow-derived dendritic cells (BMDCs) were collected from the bone marrow of male mice (C57BL/6, 4 weeks old) and cultured with granulocyte

macrophage-colony stimulating factor (GM-CSF, CK24, Novoprotein, Shanghai, China) for 3 days. Moreover, K7M2 cells were incubated with different treatments for 12 h, after which the supernatants were collected. BMDCs were incubated with these supernatants for 12 h. The maturation of BMDCs was determined via C6 plus, and matured DCs were $CD11c^+$, $CD80^+$ and $CD86^+$ (n = 3).

To promote T-cell activation, primary T cells were collected from the spleens of male mice (C57BL/6, 4 weeks old) and cultured with 1 mM sodium pyruvate solution, 55 μM of 2-mercaptoethanol, and 1 nM of HEPES solution. Moreover, K7M2 cells were incubated with different treatments for 12 h, after which the supernatants were collected. Primary T cells were incubated with these supernatants for 12 h. The number of collected cells was determined by C6 plus, and the activated T cells were $CD3^+$, $CD45^+$, and $CD4^+$/$CD8^+$/$GZMB^+$ (n = 3).

To measure IFN-γ production, K7M2 cells were incubated with different treatments for 12 h. Then the groups treated with US were further incubated for 3 h after US (30 kHz, 3W/cm^2, 3 min). The IFN-γ levels were measured with a standard IFN-γ ELISA kit (n = 3).

To assess the impact on T cell memory differentiation, primary T cells were collected from the spleens of male mice (C57BL/6, 4 weeks old) and cultured with 1 mM sodium pyruvate solution, 55 μM of 2-mercaptoethanol and 1 nM of HEPES solution. Moreover, K7M2 cells were incubated with different treatments for 12 h and co-cultured with the primary T cells for 2 days. The number

of collected cells was determined by C6 plus. The effective T memory cells were $CD3^+$, $CD8^+$, $CD44^+$, and $CD62L^-$. The central T memory cells were $CD3^+$, $CD8^+$, $CD44^+$, and $CD62L^+$ (n = 3).

In vivo toxicity evaluation

For toxicity evaluation, healthy Balb/c mice were divided into 2 groups (n = 5) for assessment of the biosafety of SHK@Mn-TiO_2. PBS and SHK@Mn-TiO_2 (10 mg/kg) were subcutaneously injected. Then, these mice were sacrificed at the same time, and the main organs (heart, liver, spleen, lung, kidney and skin) were collected in 4% paraformaldehyde solution for tissue fixation. Blood and serum were collected for routine blood examination and serum biochemistry analysis.

In vivo anti-tumor study

All experiments with specific pathogen-free (SPF) grade 4-week-old BALB/c mice and C57BL/6 mice were carried out in accordance with the guidelines approved by the Ethics Committee of Soochow University (Suzhou, China) (approval number: 202402A0105). Female Balb/c mice bearing subcutaneous K7M2 tumors ($5*10^6$) were chosen as the experimental tumor models.

When the tumor volume reached 100 mm^3, the Balb/c mice were randomized into five groups (n = 5 per group) with the following treatments: (1) control, PBS, (2) intratumoral injection of 5 mg/kg Mn-TiO_2, (3) intratumoral injection of 5 mg/kg SHK@Mn-TiO_2, (3) intratumoral injection of 5 mg/kg Mn-TiO_2 following US (30 kHz, 10 W/cm^2, 10 min) and (5) intratumoral injection of 5 mg/kg SHK@Mn-TiO_2 following US (30 kHz, 10 W/cm^2, 10 min). Injections were carried out on days 0, 2, and 4. US was carried out on days 1, 3, and 5. The tumor volume and body weight were

recorded every 2 days. The tumor volume was calculated via the following formula: width^2 × length/2. After the treatment course, the mice were sacrificed at random. The tumors were removed for histopathological analysis including H&E staining. Immunohistochemical staining (for PKM2, HK-2, HIF-1α and TUNEL) of the tumors was subsequently performed. Immunofluorescence staining ($CD4^+$ and $CD8^+$ cells) was also performed after treatment.

In vivo immune evaluation

When the tumor volume reached 100 mm^3, the Balb/c mice were randomly divided into five groups (*n* = 5 per group) with the following treatments: (1) control, PBS, (2) intratumoral injection of 5 mg/kg Mn-TiO_2, (3) intratumoral injection of 5 mg/kg SHK@Mn-TiO_2, (3) intratumoral injection of 5 mg/kg Mn-TiO_2 following US (30 kHz, 10 W/cm^2, 10 min) and (5) intratumoral injection of 5 mg/kg SHK@Mn-TiO_2 following US (30 kHz, 10 W/cm^2, 10 min). Injections were carried out on days 0, 2, and 4. US was carried out on days 1, 3, and 5. The tumor tissues, spleens and the lymph nodes near the tumor were collected on day 7 and processed into single-cell suspensions for flow cytometry detection of a variety of immune cells. The T cells were $CD3^+$, $CD45^+$ and $CD4^+$/$CD8^+$. Treg cells were $CD3^+$, $Foxp3^+$, $CD4^+$ and $CD45^+$. M1 macrophages were $CD45^+$, $CD80^+$, $CD11b^+$ and $F4/80^+$. M2 macrophages were $CD45^+$, $CD206^+$, $CD11b^+$ and $F4/80^+$. MDSCs were $CD11b^+$, $Ly6G^+$, $Ly6C^+$ and $Gr\text{-}1^+$. Mature DC cells were $CD11c^+$, $CD86^+$ and $CD80^+$. Moreover, the tumor, lymph node, and blood samples were obtained for IFN-γ and TNF-α ELISA analysis (n = 5).

In vivo tumor rechallenge experiment

Subcutaneous K7M2 tumor model mice in 60 days post-treatment with SHK@Mn-TiO_2 were injected with K7M2 ($5*10^6$) in the heterolateral subcutis. Moreover, the blood samples were obtained for effective memory T cell, central memory T cell, IFN-γ and TNF-α analysis. The Balb/c mice were divided into two or three groups (n = 5 per group) with the following treatments: (1) control, PBS, and (2) intratumoral injection of 5 mg/kg SHK@Mn-TiO_2 following US (30 kHz, 10 W/cm^2, 10 min) before or (1) control, PBS, (2) intratumoral injection of 5 mg/kg SHK@Mn-TiO_2 following US and intraperitoneal injection of 10 mg/kg XMG1.2 antibody, and (3) intratumoral injection of 5 mg/kg SHK@Mn-TiO_2 following US. When the tumor volume reached 100 mm^3, the tumor volume and body weight were recorded every 2 days. Tumor volume was calculated based on the following formula: width^2 × length/2. The tumors were removed for histopathological analysis including H&E staining. Immunofluorescence staining ($CD4^+$ and $CD8^+$ cells) was also performed after treatment.

To assess the T cell memory differentiation, blood samples were obtained. The collected cells in serum were determined by C6 plus. The effective T memory cells were $CD3^+$, $CD8^+$, $CD44^+$, and $CD62L^-$. The central T memory cells were $CD3^+$, $CD8^+$, $CD44^+$, and $CD62L^+$ (n = 5).

Statistical analysis

All the quantitative experiments were performed in triplicate unless otherwise indicated. The data are presented as the mean ± standard deviation (SD). Statistical comparisons were performed using t-test for two groups and one-way analysis of variance (ANOVA) for more than two groups Statistical differences in survival were measured by the log-rank test. The significance was

expressed with *$p < 0.05$, **$p < 0.01$, and ***$p < 0.001$. For all tests, statistical analyses were performed using Graphpad prism (version 8.0), and a two-sided $p < 0.05$ was deemed statistically significant.

2. Supporting Figures

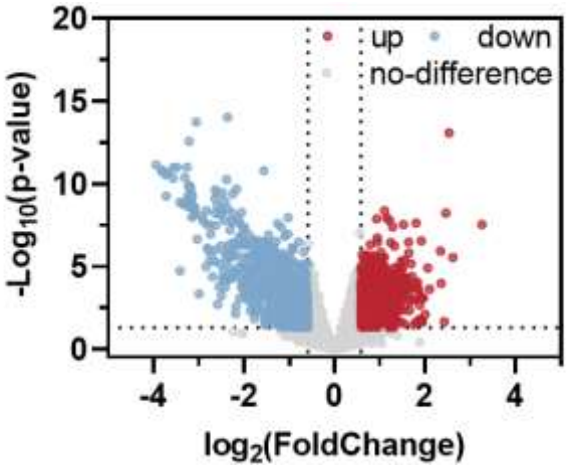

Figure S1. Volcano plot of DEGs between normal bones and OS tissues.

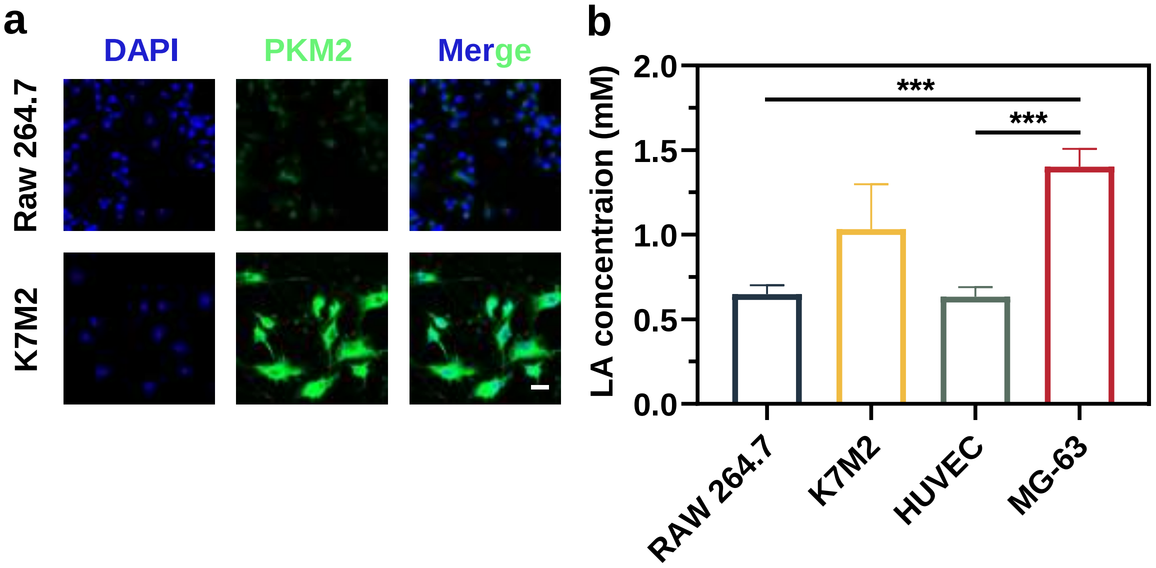

Figure S2. (a) CLSM images of PKM2-specific immunofluorescence in RAW 264.7 and K7M2 cells. Scale bar = 20 μm. (b) LA concentrations of RAW 264.7 cells, K7M2 cells, HUVEC cells, and MG-63 cells.

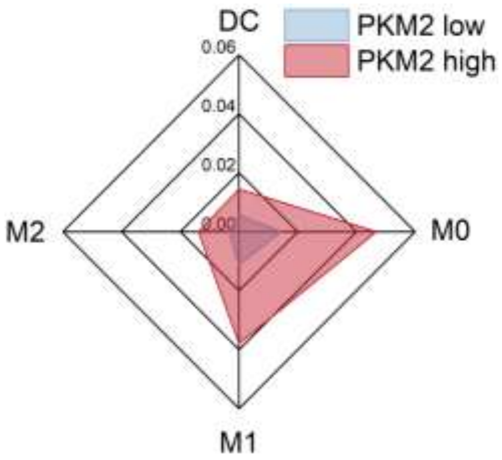

Figure S3. Estimated property of Macrophages in different OS tissues divided by PKM2 expression.

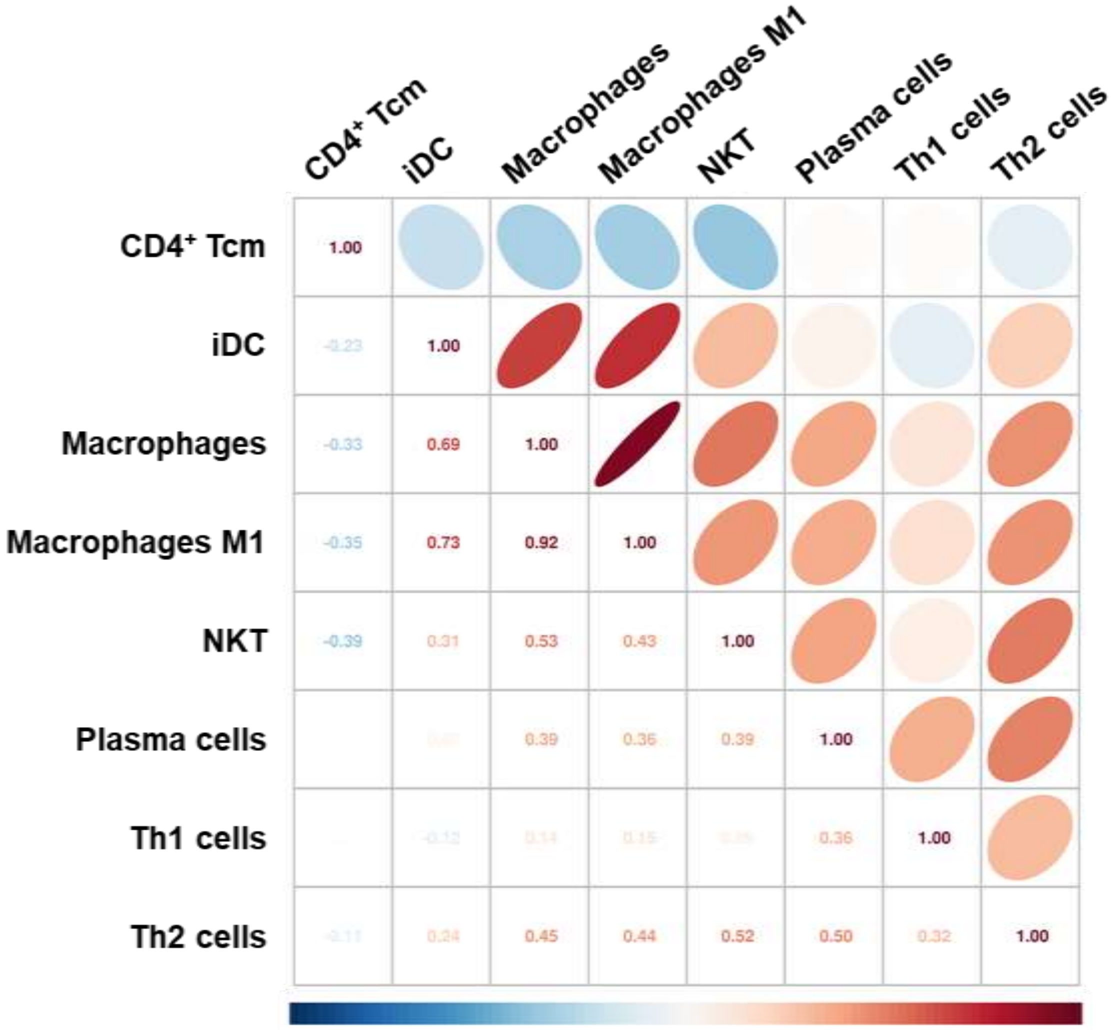

Figure S4. Correlation analysis between immune cells in OS samples with high PKM2 expression.

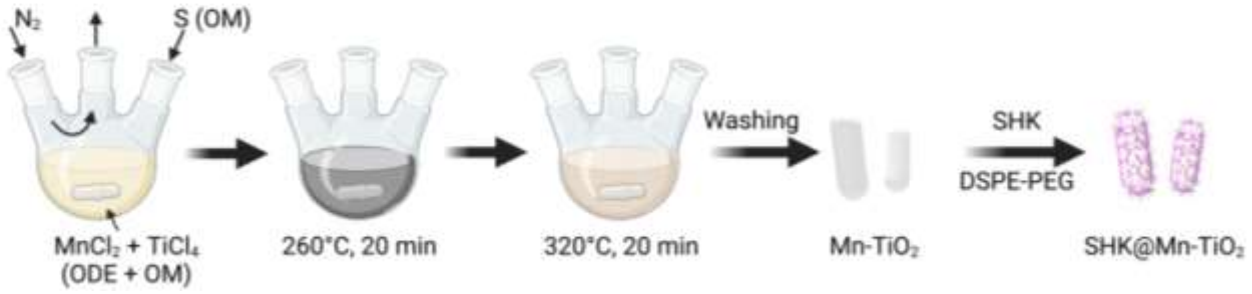

Figure S5. Schematic diagram of the synthesis and modification of SHK@Mn-TiO_2.

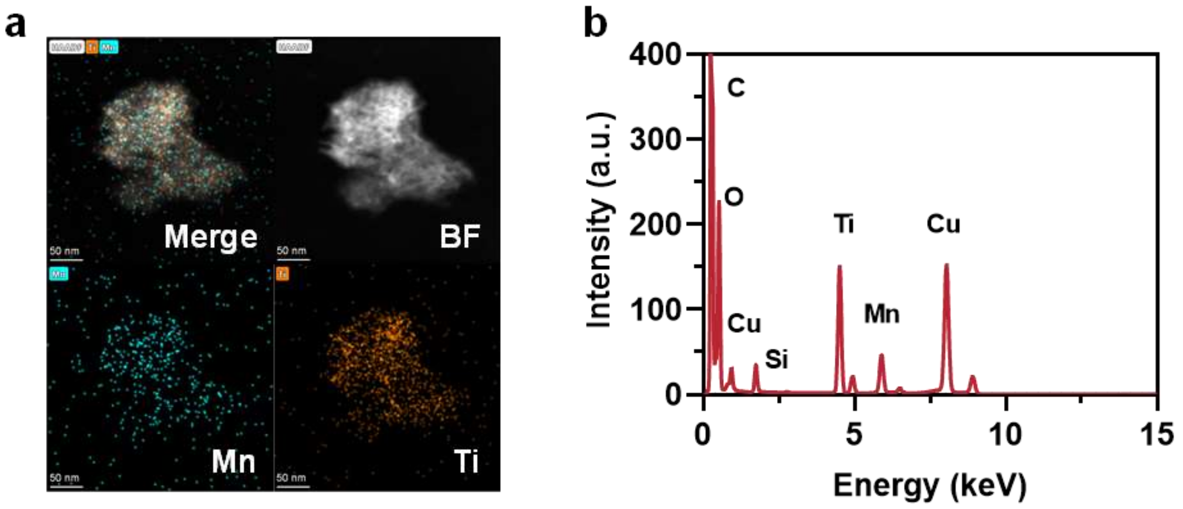

Figure S6. Element mapping images and EDS spectrum of SHK@Mn-TiO_2. Scale bar = 50 nm.

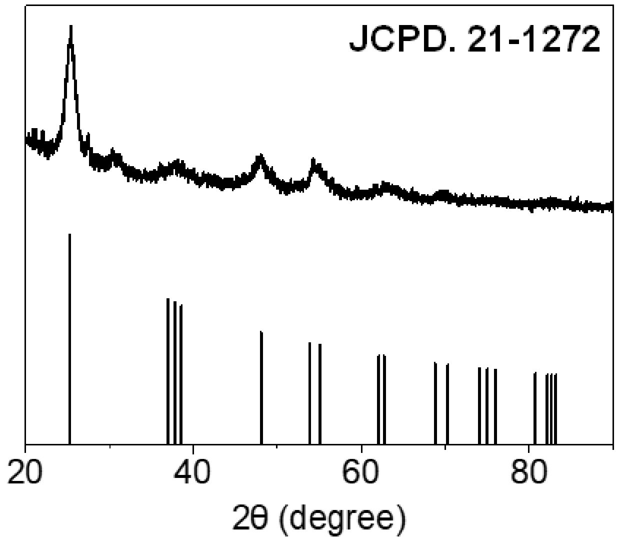

Figure S7. XRD spectrum of SHK@Mn-TiO_2.

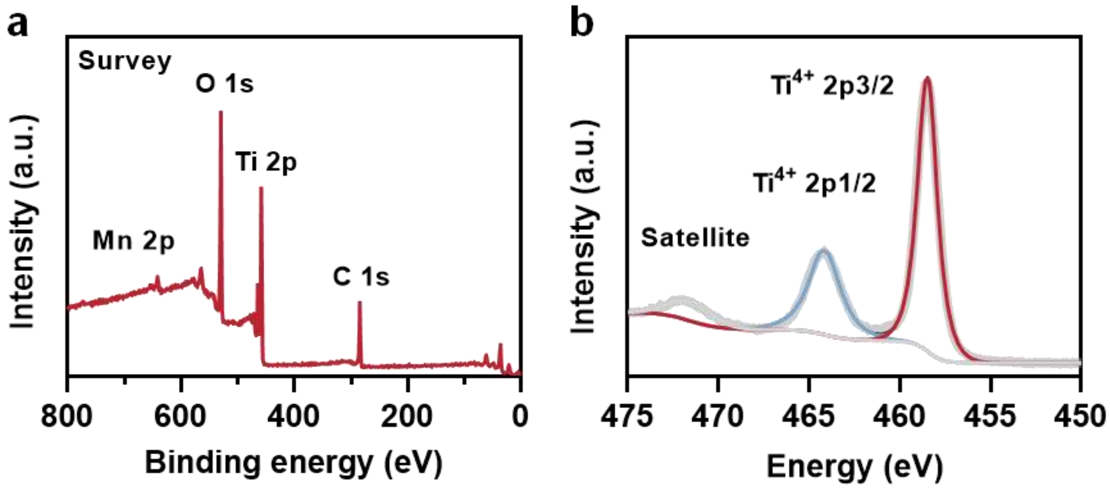

Figure S8. XPS spectra of survey (a) and Ti 2p (b).

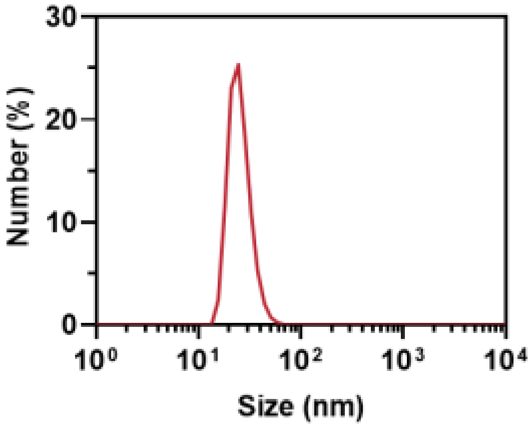

Figure S9. Hydrodynamic diameter of SHK@Mn-TiO_2.

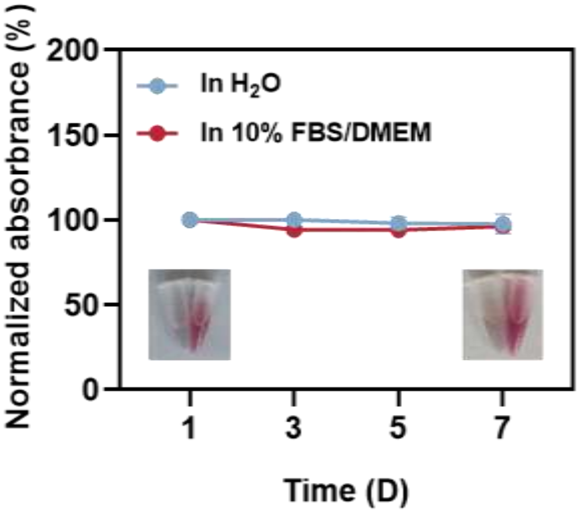

Figure S10. Stability and representative images of SHK@Mn-TiO_2.

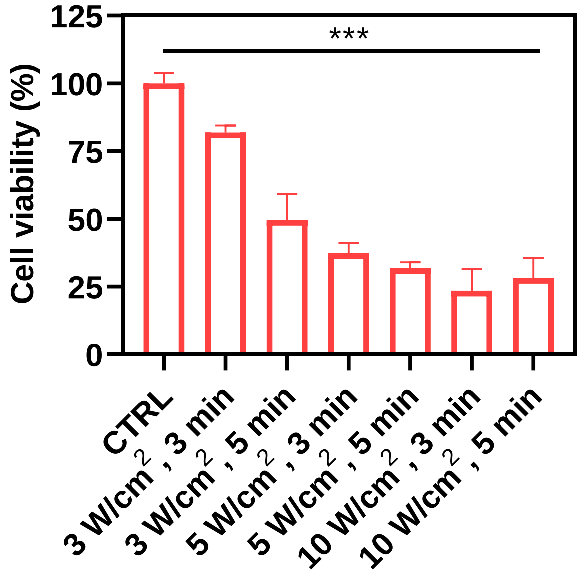

Figure S11. Relative viability of K7M2 cells dealt with different ultrasound parameters.

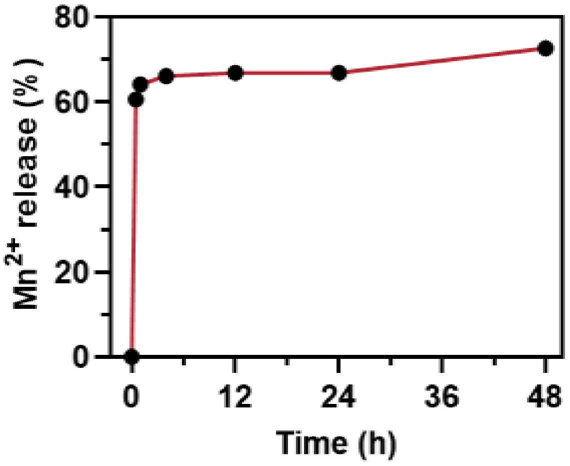

Figure S12. Mn^{2+} release of the SHK@Mn-TiO_2.

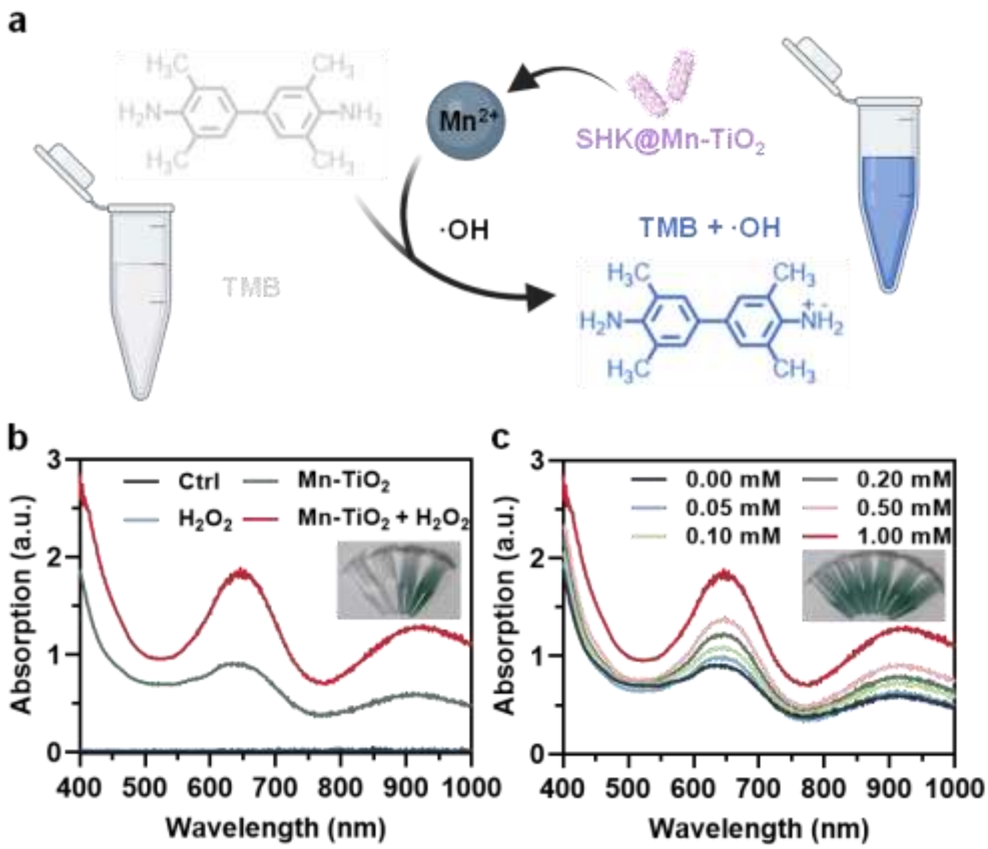

Figure S13. Fenton-like reactions of Mn-TiO$_2$. (a) Scheme of ·OH generation detection by TMB. (b) ·OH generation and representative images of Mn-TiO$_2$ incubated with different treatments. (c) H_2O_2 concentration-dependent ·OH generation and representative images of Mn-TiO$_2$ incubated with different treatments.

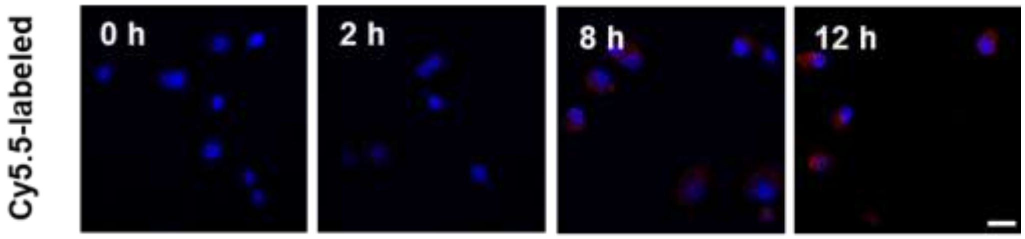

Figure S14. CLSM images of K7M2 cells incubated with Cy5.5-labeled SHK@Mn-TiO$_2$ for different time. Scale bar = 20 μm.

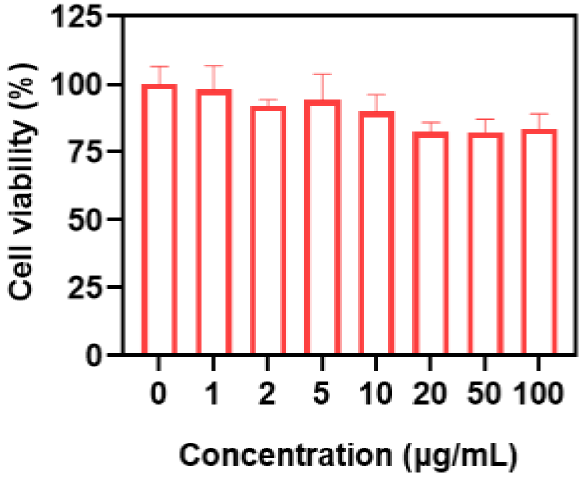

Figure S15. Relative viability of RAW 264.7 cells after treatment with different concentrations of SHK@Mn-TiO_2.

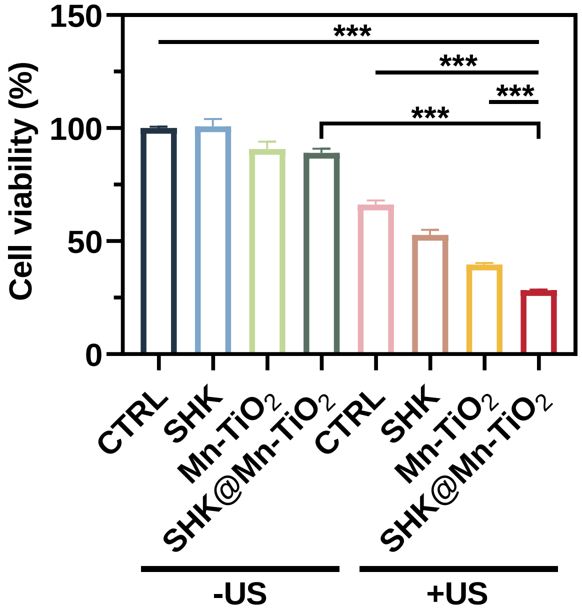

Figure S16. Relative cell viability of K7M2 cells after different treatments.

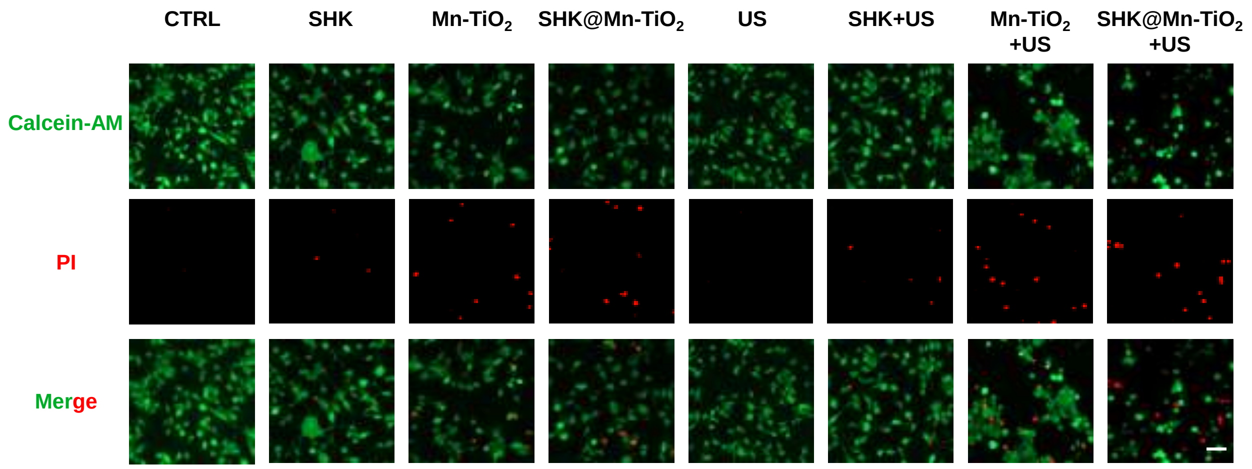

Figure S17. CLSM images of Calcein-AM/PI immunofluorescence staining after different treatments. Scale bar = 40 μm.

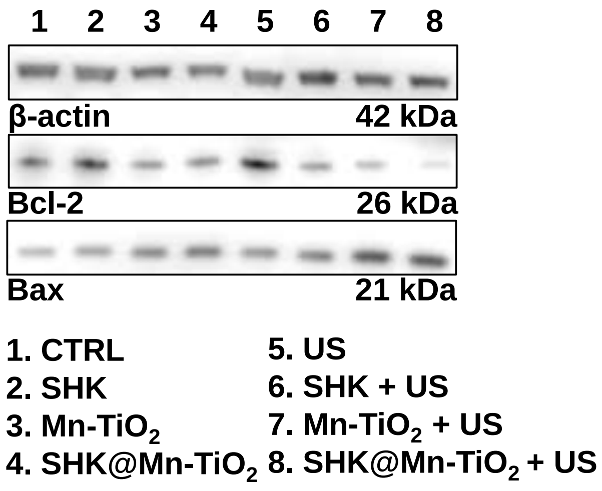

Figure S18. Western blot analysis of Bcl-2 and Bax after different treatments. Groups 1-8 represent CTRL, SHK, Mn-TiO_2, SHK@Mn-TiO_2, US, SHK + US, Mn-TiO_2 + US, and SHK@Mn-TiO_2 + US, respectively.

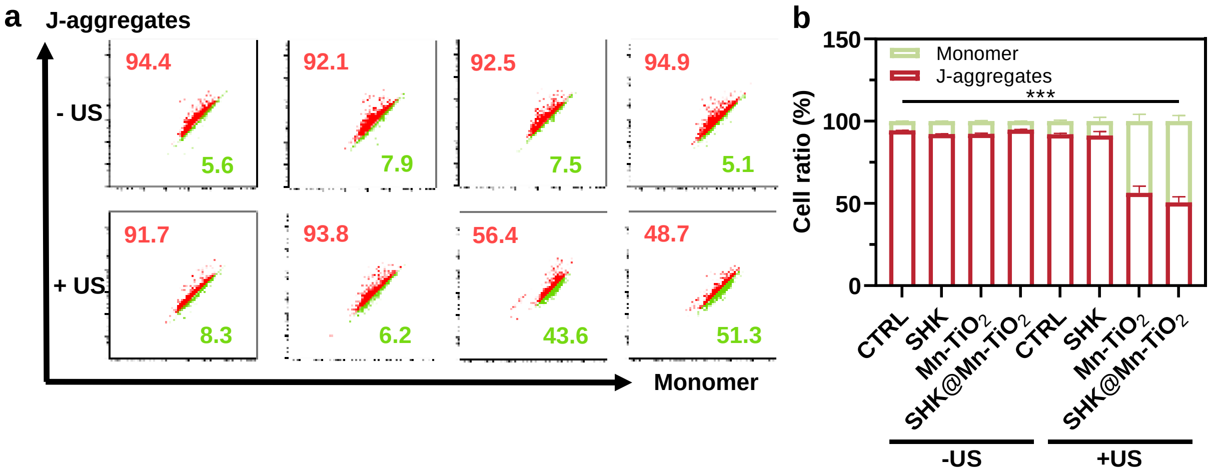

Figure S19. Representative flow dot plots (a) and quantitative analysis (b) of the membrane potential after different treatments.

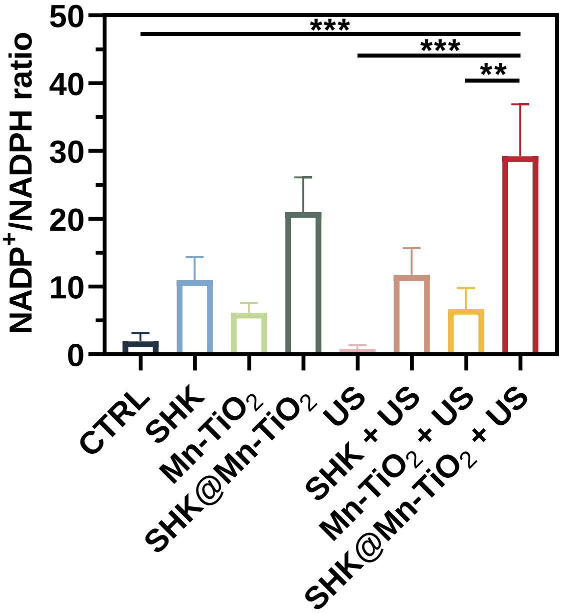

Figure S20. $NADP^+$/NAPDH ratio during oxidative phosphorylation after different treatments.

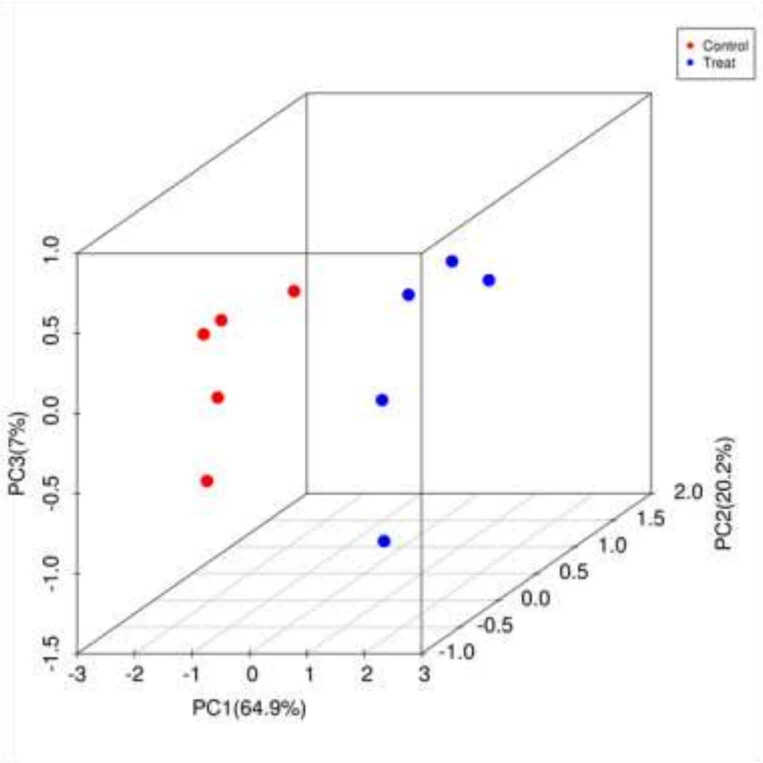

Figure S21. Score scatter plot 3D of the PCA model for the control and treatment groups.

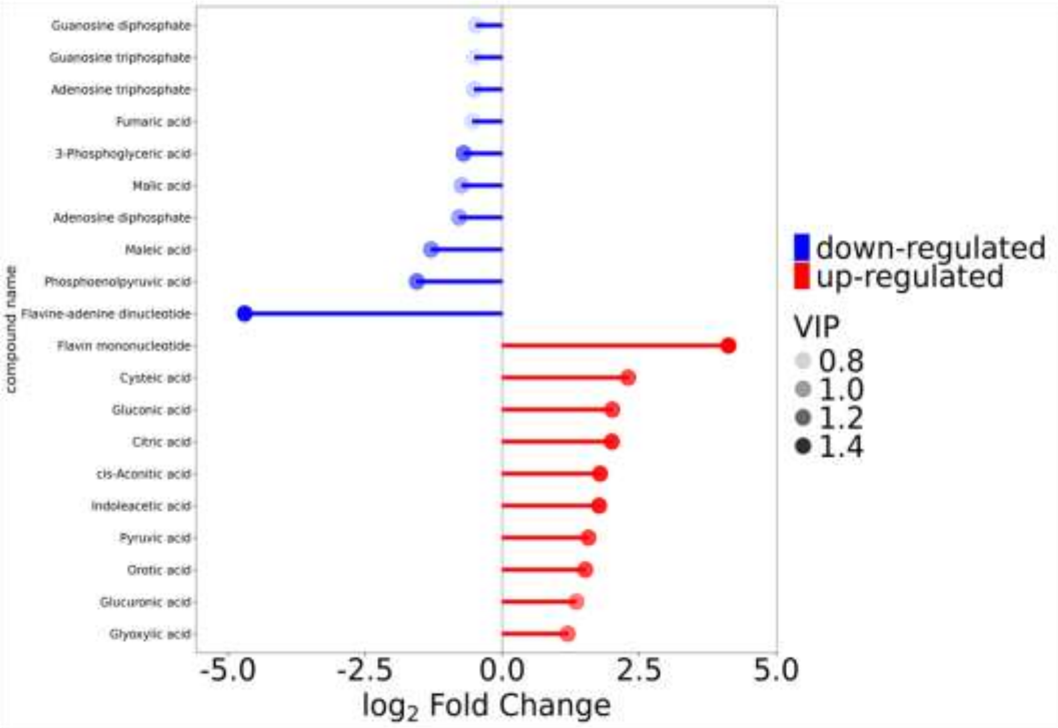

Figure S22. Matchstick analysis for the control and treatment groups.

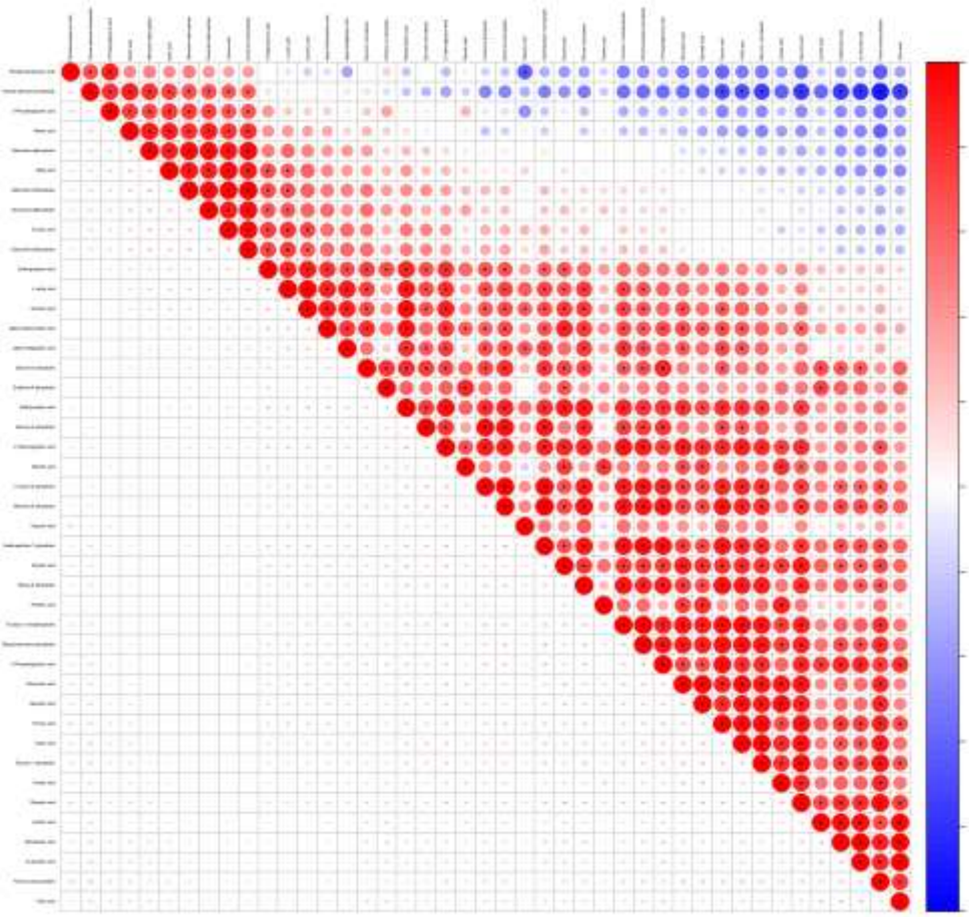

Figure S23. Heatmap of correlation analysis for the control vs treatment groups.

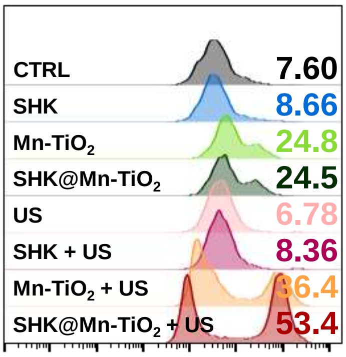

Figure S24. FACS analysis of the DCF$^+$ cell ratio after different treatments.

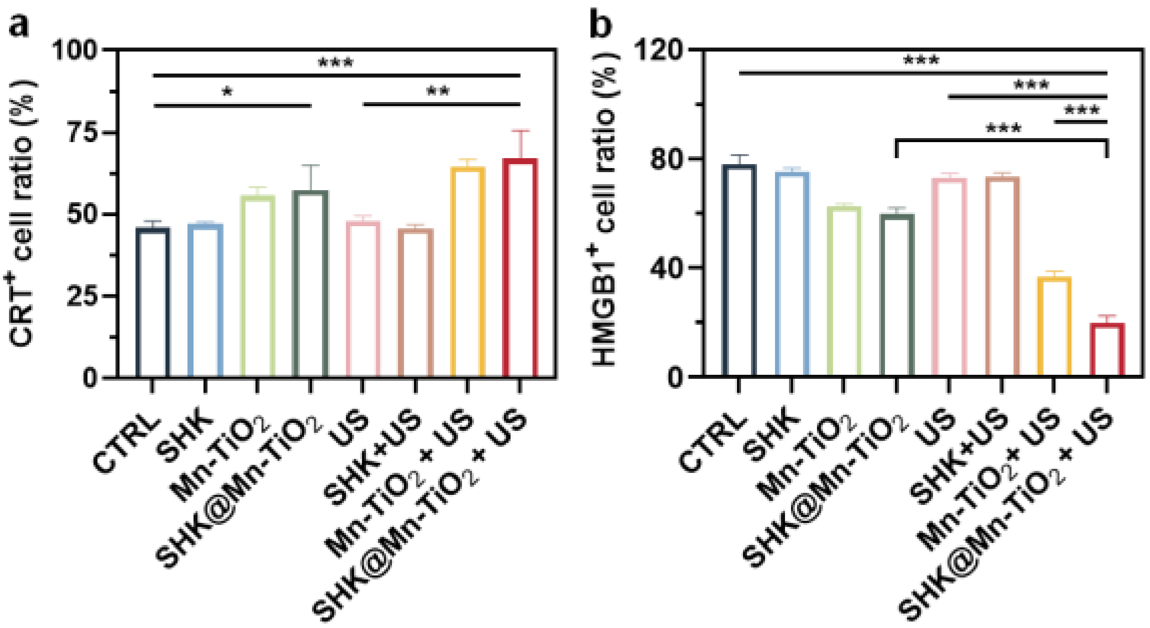

Figure S25. Quantitative analysis of the CRT^{+} (c) and HMGB1^{+} (d) cell ratios by FACS after different treatments.

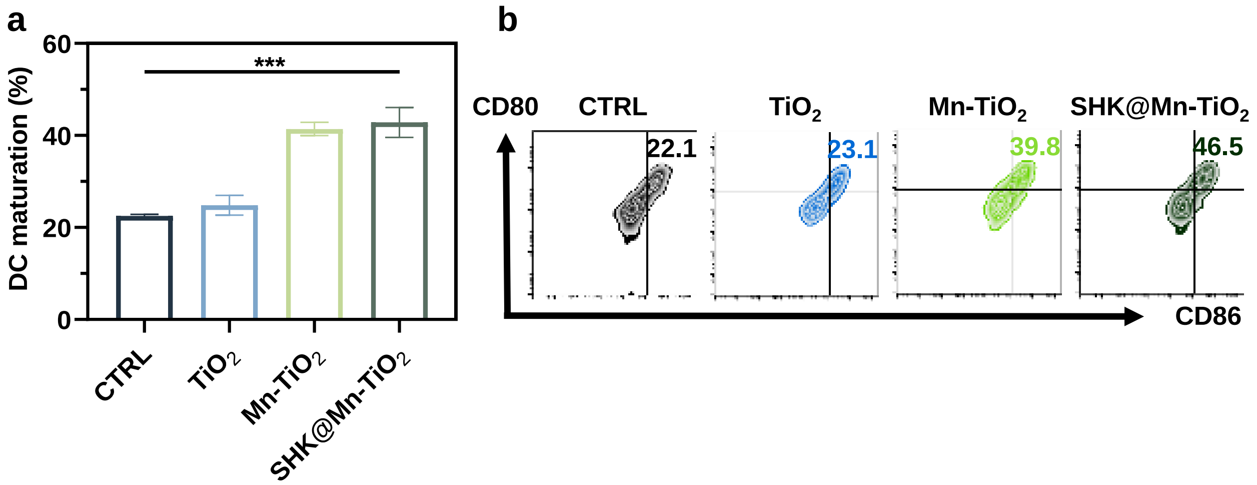

Figure S26. Quantitative analysis (a) and flow dot plots (b) of DC maturation by FACS after different treatments.

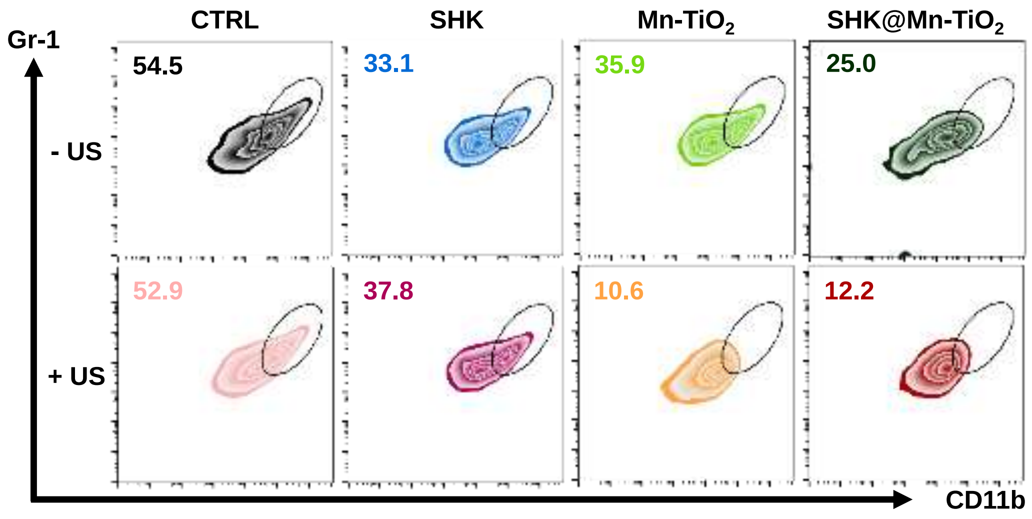

Figure S27. Flow dot plots of MDSCs by FACS after different treatments.

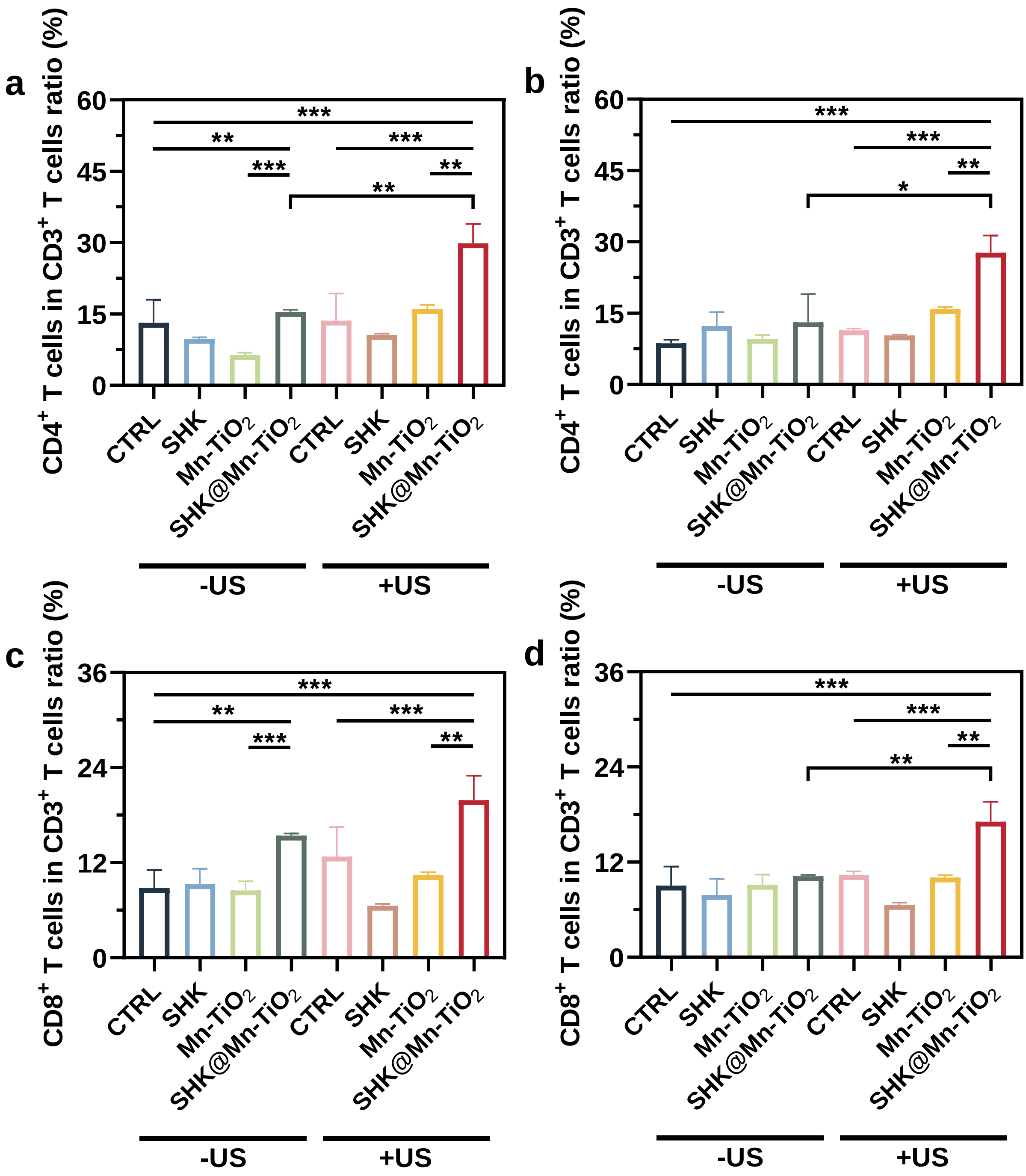

Figure S28. Repeating quantitative analysis of the ratio of CD4$^+$ (a, b) and CD8$^+$ (c, d) T cells in the CD3$^+$ T cell population by FACS after different treatments.

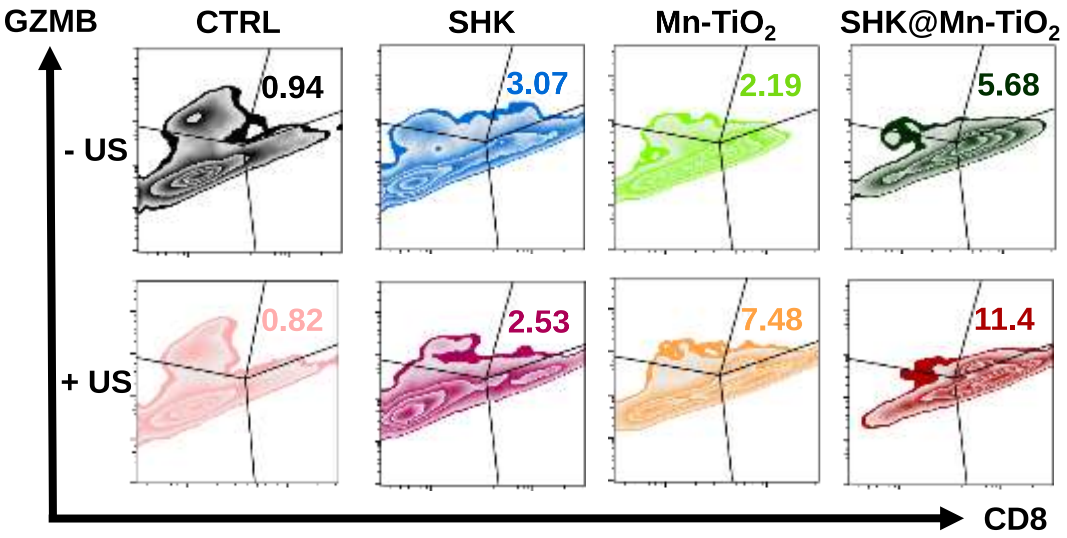

Figure S29. Flow dot plots of the ratio of $GZMB^+$ and $CD8^+$ T cells in the $CD3^+$ T cell population by FACS after different treatments.

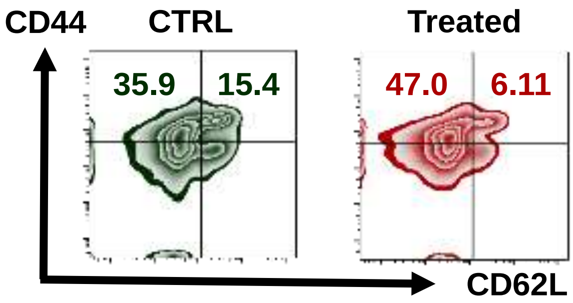

Figure S30. Flow dot plots of $CD62L^-$ $CD44^+$ Tem and $CD62L^+$ $CD44^+$ Tcm in T cells co-cultured with treated K7M2 cells.

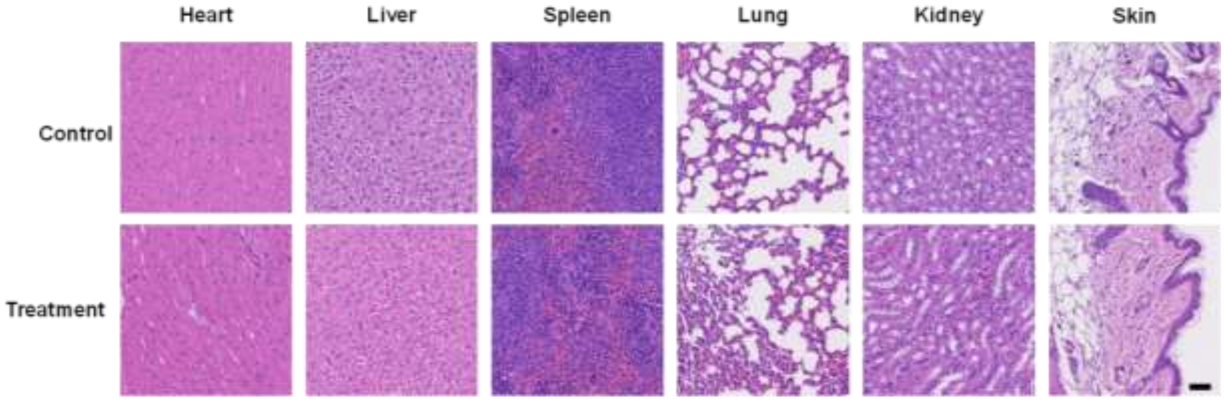

Figure S31. Representative H&E staining of the main tissues at 30 days post treatment. Scale bar = 100 μm.

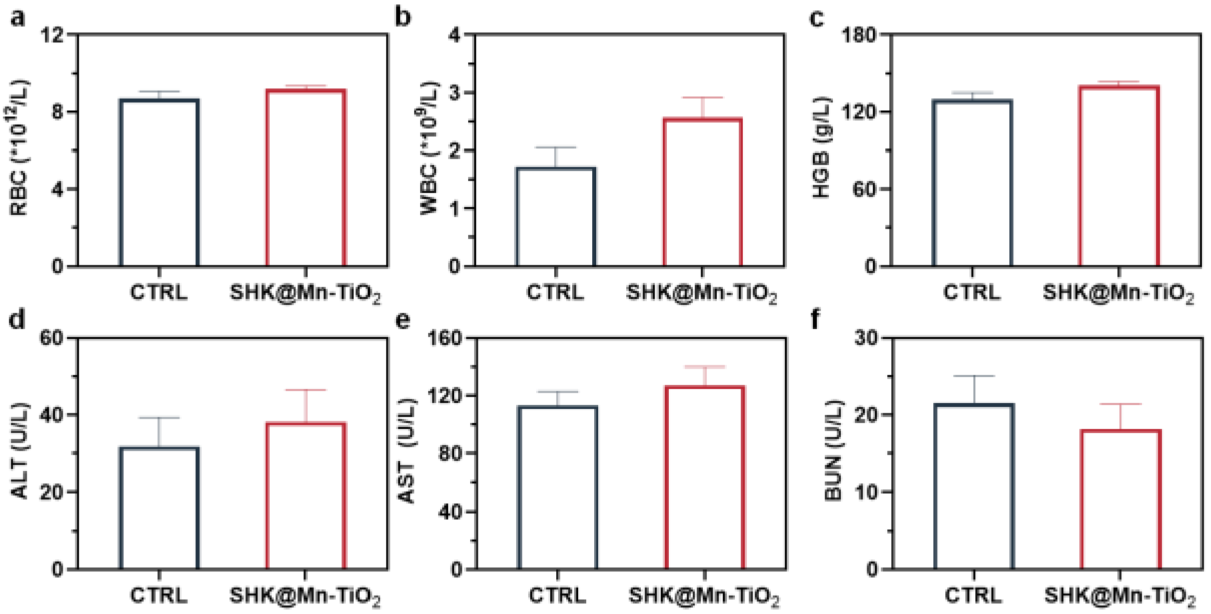

Figure S32. A complete blood count and serum biochemical analysis were performed at 30 days post treatment.

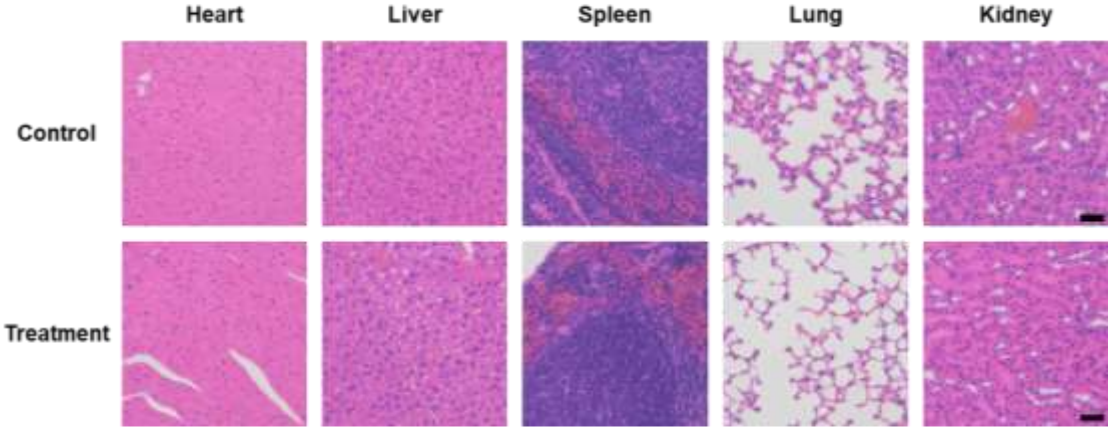

Figure S33. Representative H&E staining of the main tissues at 60 days post treatment. Scale bar = 100 μm.

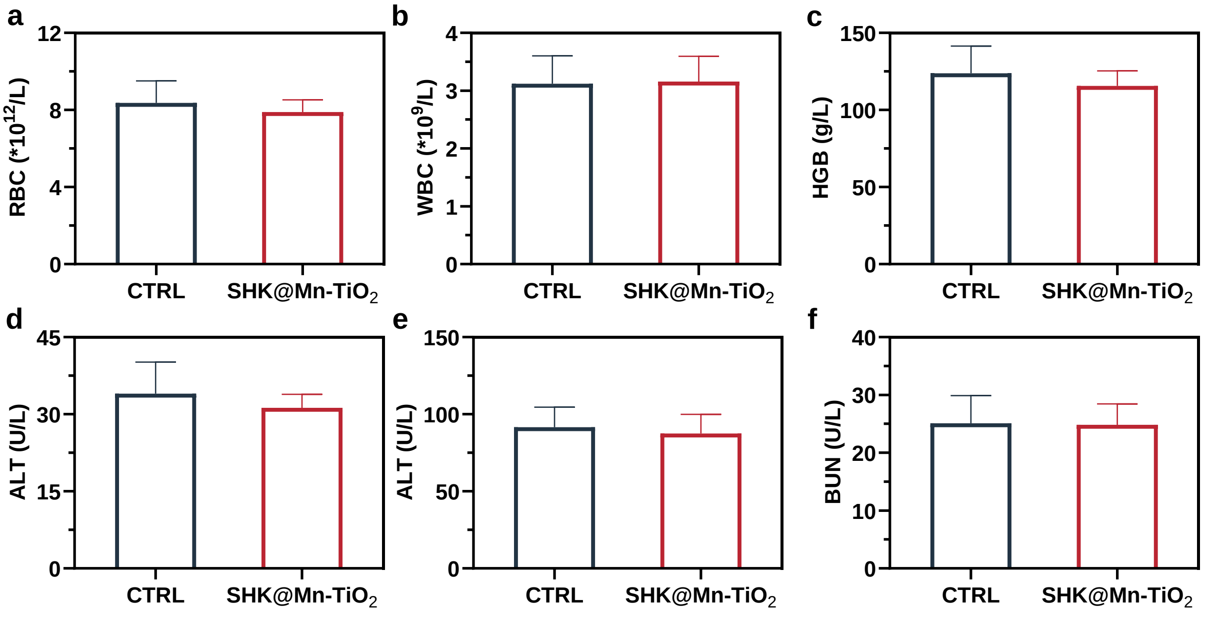

Figure S34. A complete blood count and serum biochemical analysis were performed at 60 days post treatment.

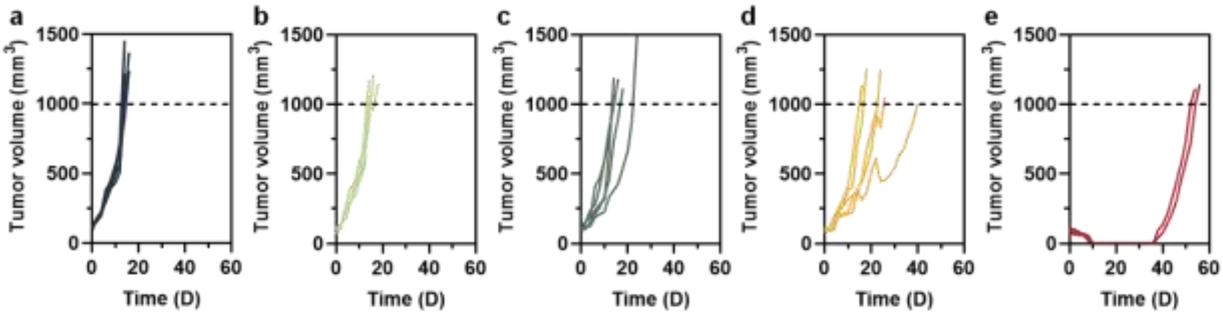

Figure S35. Detailed tumor volumes of individual mice in the CTRL (a), Mn-TiO_2 (b), SHK@Mn-TiO_2 (c), Mn-TiO_2 + US (d), and SHK@Mn-TiO_2 + US (e) groups.

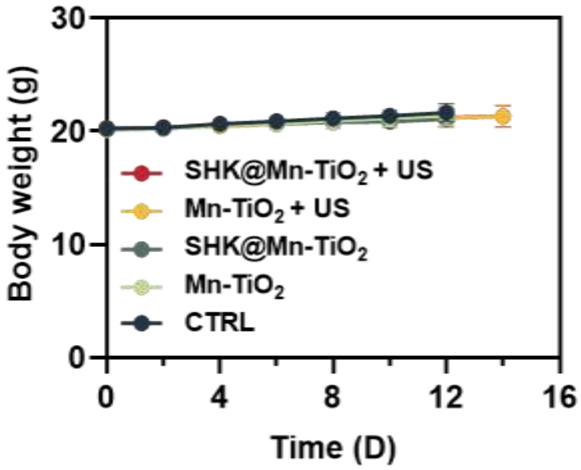

Figure S36. Body weights after different treatments.

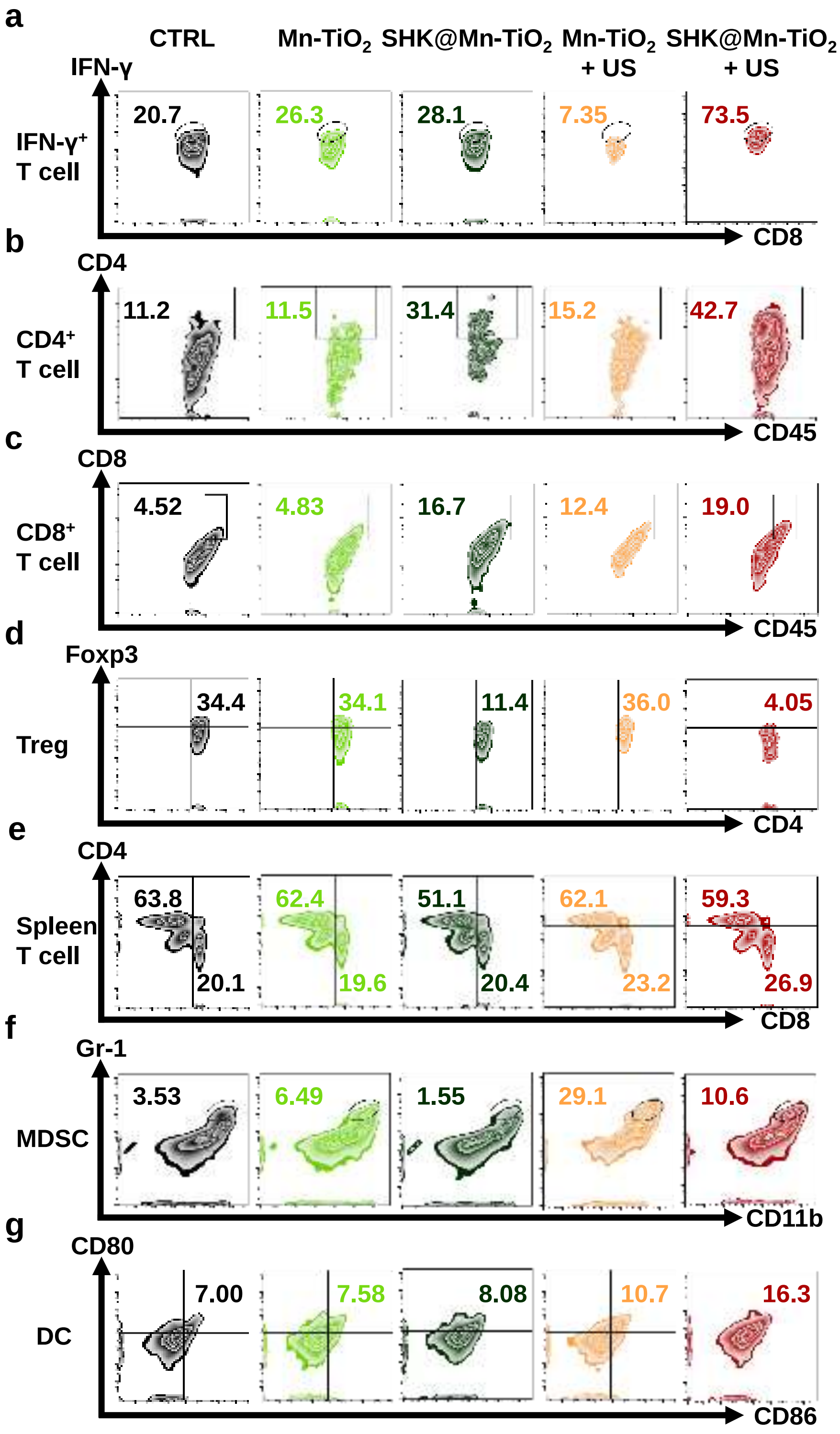

Figure S37. Flow dot plots of IFN-γ$^+$ CD8$^+$ T cells in tumor (a), CD3$^+$ CD4$^+$ T cells in tumor (b), CD3$^+$ CD8$^+$ T cells in tumor (c), CD4$^+$ Foxp3$^+$ Tregs in tumor (d), CD3$^+$ CD4$^+$/CD4$^+$ CD8$^+$ T cells in spleen (e), CD11b$^+$ Gr-1$^+$ MDSCs in tumor (f), and CD80$^+$ CD86$^+$ matured DCs in TDNLs (g) in the living tumor on 7 days post treatment.

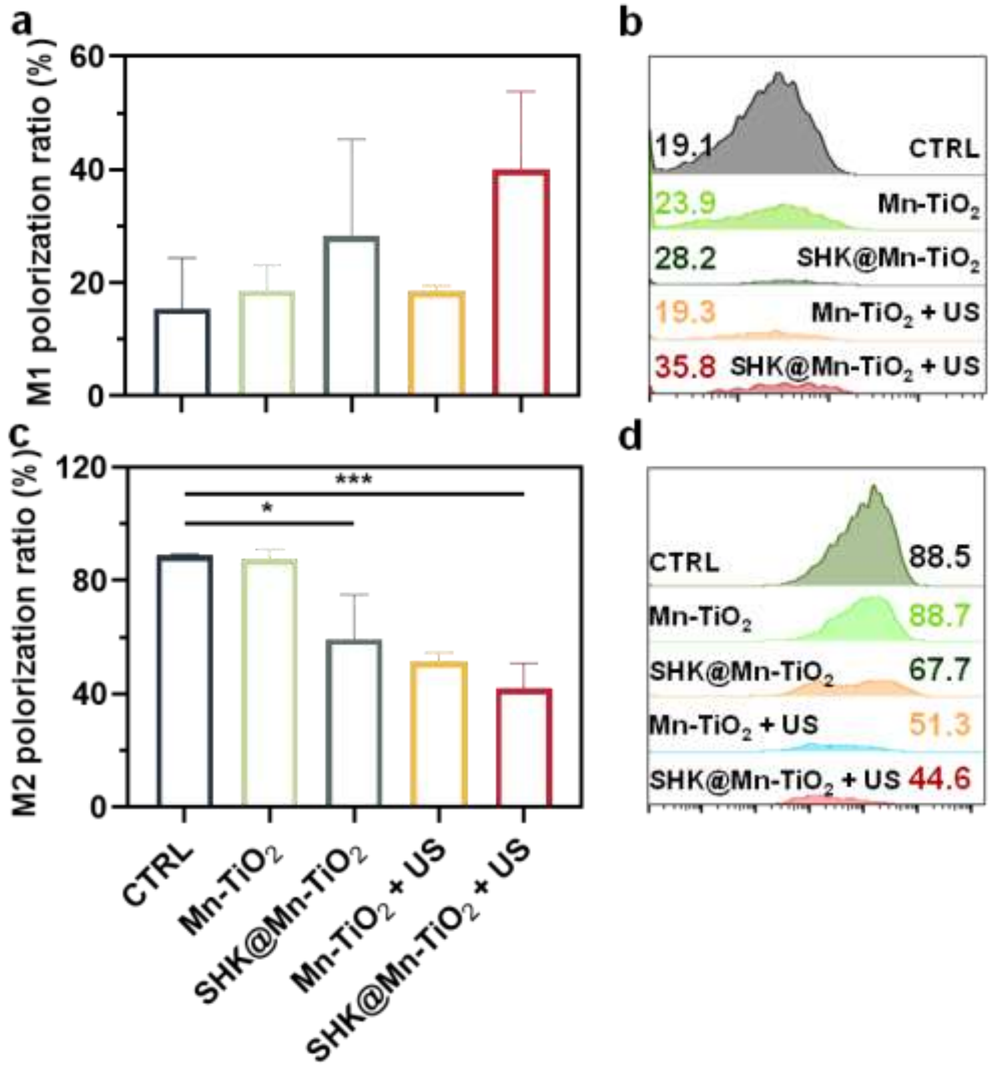

Figure S38. Tumor-associated macrophage polarization function. Quantitative analysis (a) and FACS analysis (b) of the M1 polorization ratio in the living tumors at 7 days post treatment. Quantitative analysis (c) and FACS analysis (d) of the M2 polarization ratio in living tumors on 7 days post treatment.

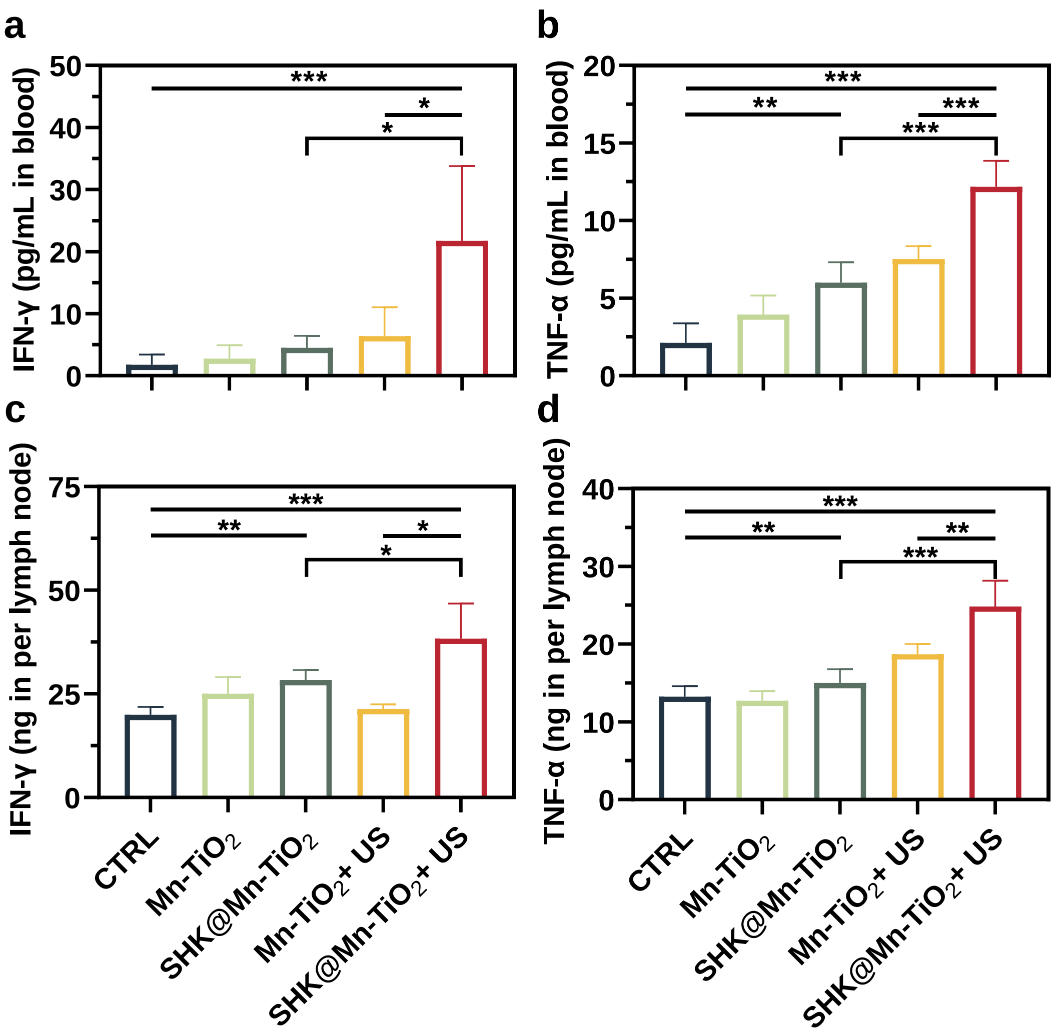

Figure S39. ELISA results of IFN-γ (a) and TNF-α (b) in blood at 7 days post-treatment. ELISA results of IFN-γ (c) and TNF-α (d) in the lymph nodes at 7 days post-treatment.

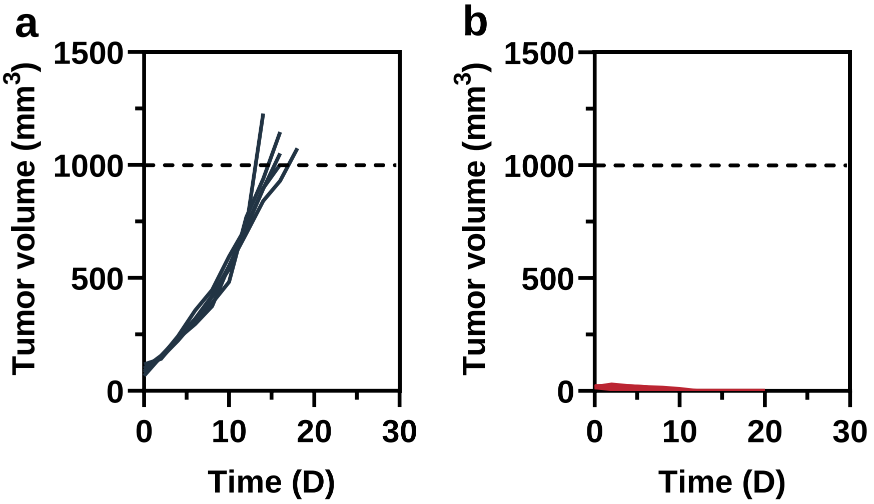

Figure S40. Detailed tumor volumes of individual mice in the control group (a) and the SHK@Mn-TiO_2 + US group (b).

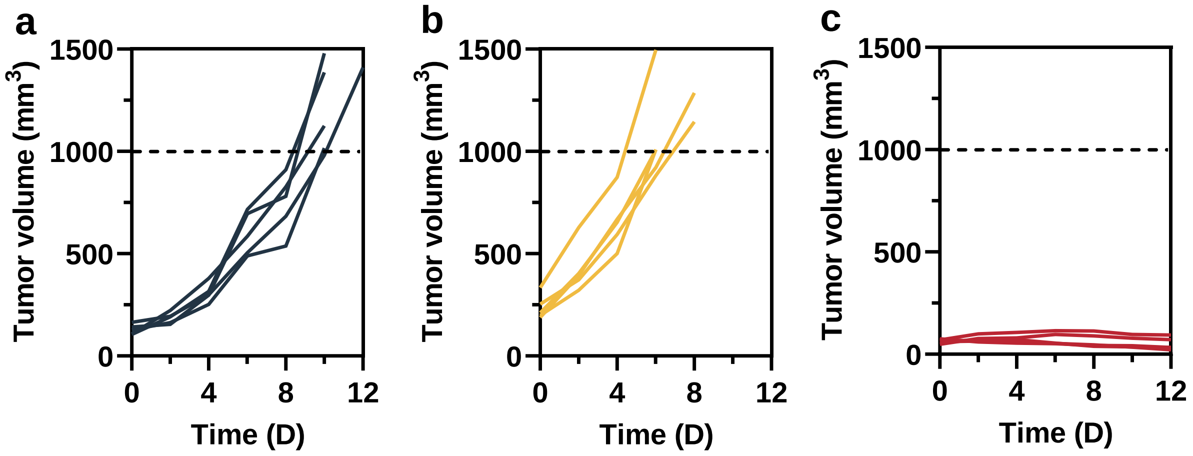

Figure S41. Detailed tumor volumes of individual mice in the control group (a), the SHK@Mn-TiO_2 + US + Anti-IFN-γ group (c), and the SHK@Mn-TiO_2 + US group (c).
